# Women's preconception health in England: a report card based on cross‐sectional analysis of national maternity services data from 2018/2019

**DOI:** 10.1111/1471-0528.17436

**Published:** 2023-03-21

**Authors:** Danielle A. J. M. Schoenaker, Judith Stephenson, Helen Smith, Kate Thurland, Helen Duncan, Keith M. Godfrey, Mary Barker, Claire Singh, Nisreen A. Alwan, for the UK Preconception Partnership

**Affiliations:** ^1^ School of Primary Care, Population Sciences and Medical Education, Faculty of Medicine University of Southampton Southampton UK; ^2^ NIHR Southampton Biomedical Research Centre University of Southampton and University Hospital Southampton NHS Foundation Trust Southampton UK; ^3^ Department of Health and Social Care Office for Health Improvement and Disparities London UK; ^4^ Elizabeth Garrett Anderson Institute for Women's Health University College London London UK; ^5^ MRC Lifecourse Epidemiology Centre University of Southampton Southampton UK; ^6^ School of Health Sciences, Faculty of Environmental and Life Sciences University of Southampton Southampton UK; ^7^ Department of Midwifery, Florence Nightingale Faculty of Nursing, Midwifery & Palliative Care King's College London London UK; ^8^ NIHR Applied Research Collaboration Wessex Southampton UK

**Keywords:** preconception health, routine health data, surveillance

## Abstract

**Objective:**

To present the first national‐level report card on the state of women's preconception health in England.

**Design:**

Cross‐sectional population‐based study.

**Setting:**

Maternity services, England.

**Population:**

All pregnant women in England with a first antenatal (booking) appointment recorded in the national Maternity Services Dataset (MSDS) from April 2018 to March 2019 (*n* = 652 880).

**Methods:**

We analysed the prevalence of 32 preconception indicator measures in the overall population and across socio‐demographic subgroups. Ten of these indicators were prioritised for ongoing surveillance based on modifiability, prevalence, data quality and ranking by multidisciplinary UK experts.

**Results:**

The three most prevalent indicators were the proportion of the 22.9% of women who smoked 1 year before pregnancy who did not quit smoking before pregnancy (85.0%), those who had not taken folic acid supplementation before pregnancy (72.7%) and previous pregnancy loss (38.9%). Inequalities were observed by age, ethnicity and area‐based deprivation level. The ten indicators prioritised were not taking folic acid supplementation before pregnancy, obesity, complex social factors, living in the most deprived areas, smoking around the time of conception, overweight, pre‐existing mental health condition, pre‐existing physical health condition, previous pregnancy loss and previous obstetric complication.

**Conclusions:**

Our findings suggest important opportunities to improve the state of preconception health and reduce socio‐demographic inequalities for women in England. In addition to MSDS data, other national data sources that record further and possibly better quality indicators could be explored and linked to build a comprehensive surveillance infrastructure.

## Abstract 2

**Linked article:** This article is commented on by Mairead Black, pp. 1196 in this issue. To view this mini commentary visit https://doi.org/10.1111/1471‐0528.17483.

## 1 INTRODUCTION

The health, behaviours and wider circumstances of women and men of reproductive age influence their own future health, are key determinants of a healthy pregnancy, and can have far‐reaching consequences for the health and development of the next generation.^1^, ^2^ The importance of optimal preconception health is recognised in many national and international policies and guidelines.^3^ These offer clinical guidance on providing preconception care to individuals planning pregnancy,^4^, ^5^ and call for population‐level efforts to improve the health of women and men of reproductive age more broadly.^6^, ^7^, ^8^

In the UK, the number of initiatives and calls for action to improve preconception health is growing.^6^, ^9^, ^10^, ^11^ As a result, awareness of the importance of preconception health among policy makers, health professionals and the community is likely to increase, and further interventions that promote pregnancy planning and preparation are likely to be developed. To inform and evaluate existing and new initiatives, and to track progress towards optimising and reducing inequalities in preconception health, there is a need for regular monitoring of the state of preconception health in England.

Following publication of the 2018 *Lancet* Series on preconception health,^1^, ^2^, ^12^ the UK Preconception Partnership^13^ proposed an annual report card to describe the state of, and trends in, preconception health using routine national data sources.^14^ It outlined a framework for reporting and monitoring of preconception health in England, which would serve to translate the compelling evidence on the importance of preconception health into policy and practice, and hold relevant organisations to account for improving the nation's preconception health and narrowing health inequalities.^14^

To inform annual reporting of preconception health, a review of national and international preconception guidelines, recommendations, position statements and policy reports was conducted in 2021 to identify preconception indicators.^3^ Indicators are defined as medical, behavioural and social risk factors or exposures, as well as wider determinants of health, that may impact potential future pregnancies among all women and men of reproductive age.^3^ Our review identified a set of 65 indicators across 12 domains that could be measured using existing core data sources in England.^3^ We proposed that the next steps to inform national surveillance should include analysis of relevant datasets to obtain national prevalence estimates of preconception indicators already routinely measured, and prioritisation of a reduced set of indicators (or core metrics) for ongoing surveillance.^3^

In England, the government Office for Health Improvement and Disparities (OHID) has a comprehensive public health surveillance system in place (Public Health Profiles).^15^ As part of the Child and Maternal Health Profile, this includes two of the indicators identified in our review^3^ (i.e. folic acid supplementation before pregnancy and obesity in early pregnancy).^16^ A wider set of potential indicators is available in the Maternity Services Dataset (MSDS), a key national data source which records annual data on all pregnant women in England. Of the 65 indicators previously identified in our review,^3^ 23 indicators are routinely recorded in the national MSDS across seven domains (wider determinants of health, emotional and social health and support, reproductive health and family planning, health behaviours and weight, mental health conditions, physical health conditions, genetic risk). The MSDS does not currently include preconception indicators related to healthcare, environmental exposures, cervical screening, immunisation and infections, and medication.

Here we present the first national‐level report card on the state of preconception health of women in England based on all indicators routinely recorded in the MSDS. We also define ten initial priority indicators for ongoing national surveillance. We present this work as initial key steps and discuss suggested actions to further develop a comprehensive surveillance infrastructure for preconception health.

## 2 METHODS

### 2.1 Study design and population

In this national population‐based study we used data from the national MSDS version 1.5 for the period 1 April 2018 to 31 March 2019. The MSDS is an administrative dataset used by providers of maternity care for clinical and service planning purposes, and by OHID for surveillance purposes.^17^, ^18^ It routinely collects patient‐level data at key stages of the maternity service care pathway in UK National Health Service (NHS)‐funded maternity services, from the first antenatal (booking) appointment until mother and baby(s) are discharged from maternity services. Data are collected through web‐based manual data entry forms completed by midwives and other healthcare professionals at every maternity unit based on discussion with pregnant women and submitted to NHS Digital. All NHS‐funded maternity units are expected to submit a set of mandatory, required and optional data items.^18^

Our study population included all women in England who attended a booking appointment during the study period (*n* = 652 880). Participant consent was not required for this study under the Health and Social Care Act 2012. Anonymised MSDS data were accessed through OHID, who have a data sharing agreement with NHS Digital. Ethics approval for the current study was granted by the University of Southampton Faculty of Medicine Ethics Committee (ID 57993) and the NHS Health Research Authority and Research Ethics Committee (IRAS ID 285601; REC reference 20/WM/0231).

### 2.2 Indicator data and definitions

Data recorded at the booking appointment were used. Data on all relevant preconception indicators as identified in our previous review^3^ and recorded in the MSDS^17^ were included for the current study (Table S1). Of the 65 indicators and 117 indicator measures identified in our review and measured in existing national data sources, 23 and 32 were recorded in the MSDS, respectively. A list of indicators identified in our previous review^3^ but not recorded in the MSDS^17^ can be found in Table S2.

Some data were ascertained retrospectively for factors concerning health and behaviours before pregnancy, such as pregnancy history, folic acid supplement use, smoking status and past medical diagnoses. Other relevant factors reflect women's characteristics at the time of the booking appointment (recommended to take place by 10 weeks' gestation^19^), such as weight status and wider determinants of health. These factors are likely to apply to women's characteristics and behaviours around the time of conception and prior to pregnancy.^20^, ^21^ Although most data are self‐reported, this varies between and within Trusts, for example for weight and height that may be measured and for previous pregnancy complications that may be checked against previous medical records.

Data recorded on alcohol consumption (4.1% of women with valid data reported consuming any alcohol at booking; 39% missing data) and substance use (1.4% of women with valid data reported using any substance; 20% missing data) were of low quality and substantially underreported and are therefore not presented in this report card.

### 2.3 Priority preconception indicators for national surveillance

To reduce preconception indicator measures to a manageable number for this first report card and for ongoing annual surveillance, we devised criteria based on prevalence and potential modifiability of factors and data quality (missing data proportion) and applied these to the 32 measures identified in our review and recorded in the MSDS. Fifteen indicators met the criteria of potentially being modifiable, prevalence of at least 5% in the overall study population, and less than 30% missing data. The second step involved a ranking exercise among members of the UK Preconception Partnership (a multi‐disciplinary group of individuals and organisations representing research, clinical practice, policy and the public).^13^ All members received an email (January 2022) with details on definitions of the 15 indicators asking them to rank these from 1 (most important) to 10 (least important) according to their importance for inclusion in national surveillance of preconception health. Of 44 active members, 27 (61%) responded. Scores were reversed and mean scores calculated to rank indicator measures.

### 2.4 Statistical analysis

The prevalence of each preconception indicator measure (unadjusted) was described for the overall population and across four subgroups of sociodemographic characteristics: maternal age, ethnicity, area‐based level of deprivation (based on postcode and expressed as the Index of Multiple Deprivation) and previous pregnancy. Adjusted prevalence estimates across subgroups were calculated for the ten identified priority indicator measures. Prevalence estimates were adjusted for the four subgroup variables to determine whether these explained differences across subgroups. The proportion of missing data for each indicator measure was described for the overall population and across subgroups.

Patients were not involved in the development of the research.

## 3 RESULTS

Women had a mean age of 30 years (SD 5.7) at their booking appointment, with a median gestational age of nine weeks and five days (interquartile range 59–80 days) (57.8% within the recommended 10 weeks' gestation). A total of 37.9% of women were pregnant for the first time.

### 3.1 Prevalence of preconception indicators

The overall prevalence of indicators ranged from 0.2% (pre‐existing hepatitis B and cancer) to 85.0% (proportion of smokers who did not quit smoking during the year before pregnancy) (Table 1).

**TABLE 1:** Overall prevalence of preconception indicators routinely recorded in the 2018/19 Maternity Services Dataset, *n* = 652 880.

Indicators^a^	%	*n*
Wider determinants of health		
Ethnic minority (*n* = 549 552)	22.8	125 099
Unemployed and seeking work (*n* = 472 181)	5.7	26 849
Living in the most deprived areas (bottom 10%)^b^ (*n* = 652 880)	14.2	92 528
Complex social factors^c^ (*n* = 527 591)	12.9	67 887
English not first language (*n* = 497 644)	20.5	101 988
Emotional and social health and support		
No adequate support available during and after pregnancy (*n* = 449 884)	5.9	26 590
Reproductive health		
Advanced maternal age at booking (≥35 years) (*n* = 652 871)	21.4	139 661
Teenage pregnancy (<20 years) (*n* = 652 871)	3.8	24 675
Known previous obstetric complication (*n* = 329 228)^d^ ^,^ ^e^	24.5	80 694
Previous pre‐eclampsia, HELLP, eclampsia, gestational proteinuria (*n* = 329 228)^e^	1.1	3469
Previous gestational hypertension (*n* = 329 228)^e^	1.6	5208
Previous gestational diabetes mellitus (*n* = 329 228)^e^	2.3	7506
Previous caesarean section (*n* = 306 430)^e^	22.8	69 990
Previous pregnancy loss (*n* = 301 168)^e^	38.9	117 258
Health behaviours and weight		
Not taking folic acid supplementation before pregnancy (*n* = 488 987)	72.7	355 648
Smoking around conception (*n* = 604 514)	19.5	117 602
Smokers who did not quit smoking during year before pregnancy (*n* = 138 422)^f^	85.0	117 602
Underweight at booking (BMI <18.5 kg/m^2^) (*n* = 496 331)	3.1	15 346
Overweight at booking (BMI 25–29.9 kg/m^2^) (*n* = 496 331)	28.0	138 774
Obesity at booking (BMI ≥30 kg/m^2^) (*n* = 496 331)	22.3	110 628
Known pre‐existing health conditions		
Mental health condition (*n* = 652 880)	9.3	60 973
Physical health condition^g^ (*n* = 652 880)	19.1	124 705
At least one mental or physical health condition (*n* = 652 880)	24.3	158 839
Diabetes (*n* = 652 880)	1.0	6343
Hypertension (*n* = 652 880)	1.0	6696
Cardiac disease (*n* = 652 880)	0.8	5184
Thromboembolic condition (*n* = 652 880)	0.6	3885
Renal disease (*n* = 652 880)	0.8	5126
Hepatitis B (*n* = 652 880)	0.2	998
Cancer (*n* = 652 880)	0.2	1085
Known family history		
Inherited condition (*n* = 652 880)	2.0	13 323
Diabetes (*n* = 652 880)	20.6	134 398

Abbreviations: BMI, body mass index; HELLP, haemolytic anaemia, elevated liver enzymes and low platelet count.

^a^ Definitions of indicators can be found in Table S1.

^b^ Based on women's postcode and the index of multiple deprivation (IMD) 2015.

^c^ Complex social factors: women who are aged under 20, experience domestic abuse, are recent migrants, asylum seekers or refugees, have difficulty reading or speaking English and/or misuse substances (alcohol and/or drugs).

^d^ Any of the following obstetric complications (*n* = 18): severe pre‐eclampsia requiring pre‐term birth, haemolytic anaemia, elevated liver enzymes and low platelet count (HELLP), eclampsia, gestational proteinuria, gestational diabetes mellitus, gestational hypertension, caesarean section, puerperal psychosis, liver cholestasis of pregnancy, antepartum haemorrhage, postpartum haemorrhage, feto‐maternal haemorrhage, antenatal/postpartum thromboembolic condition, placental abruption, uterine rupture, retained placenta requiring manual removal in theatre, extensive vaginal, cervical, or 3rd or 4th degree perineal trauma, amniotic fluid embolism.

^e^ Previous pregnancy complications among women with a previous pregnancy.

^f^ Preconception smoking cessation among women who smoked 12 months before pregnancy.

^g^ Any of the following physical health conditions (*n* = 17): diabetes, hypertension, cardiac disease, thromboembolic condition, renal disease, hepatitis B, cancer, gynaecological conditions, gastrointestinal condition, respiratory disease, endocrine condition, musculoskeletal condition, central nervous system condition, haematological condition, autoimmune condition, infectious hepatitis A, hepatitis C.

**FIGURE 1:**
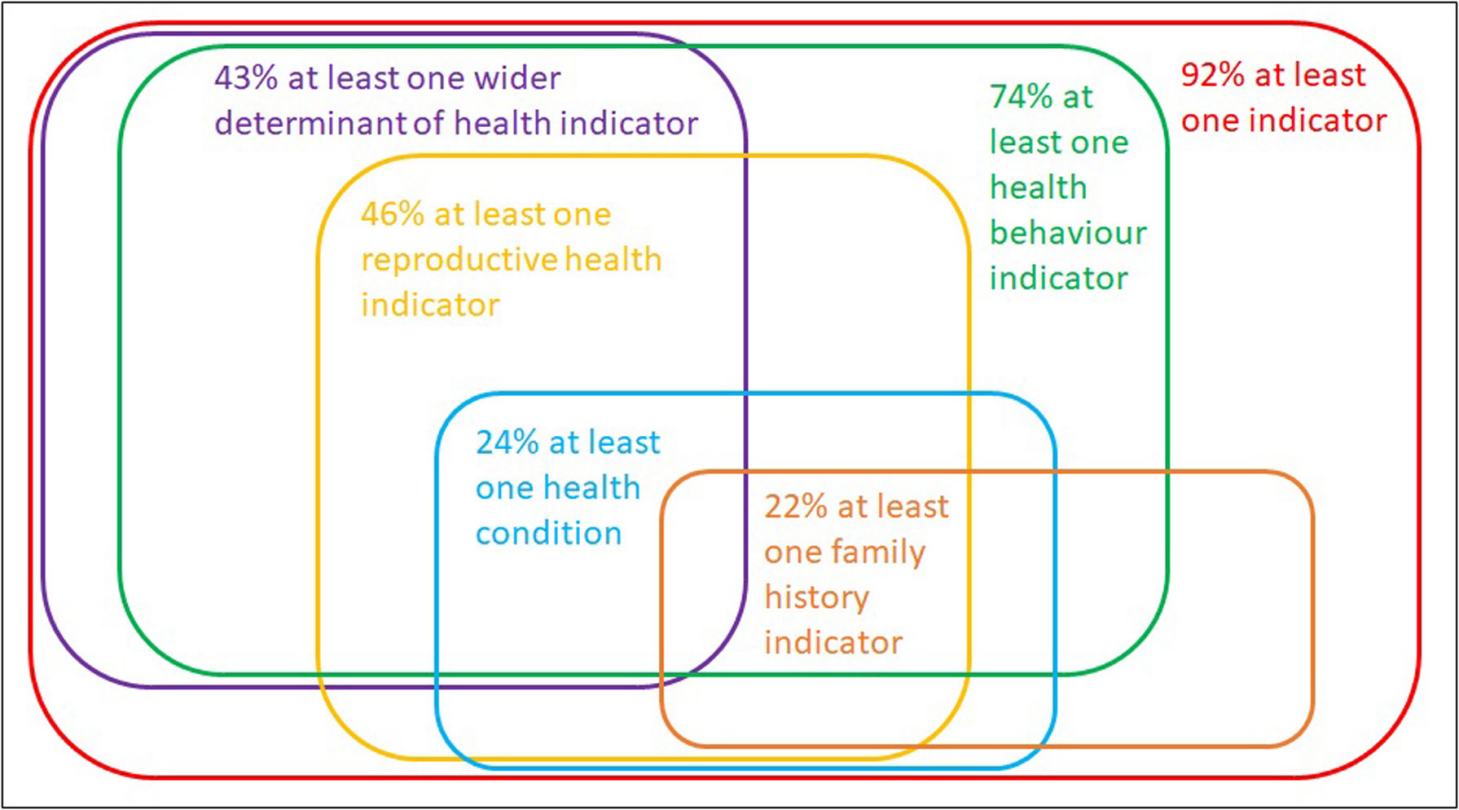
Prevalence of preconception indicator domains among pregnant women in England. The size of each area and the size of their overlap are an approximation and do not accurately reflect the percentages. Domains: wider determinants of health (indicators: ethnic minority, unemployed and seeking work, living in the most deprived areas, complex social factors and/or English not the first language); reproductive health (indicators: advanced maternal age, teenage pregnancy, previous obstetric complication and/or previous pregnancy loss); health behaviours and weight (indicators: not taking folic acid supplementation before pregnancy, smoking around the time of conception, not quitting smoking during year before pregnancy, underweight, overweight and/or obesity); health conditions (indicators: pre‐existing mental health condition and/or pre‐existing physical health condition); family history (indicators: family history of inherited condition and/or family history of diabetes).

The prevalence of indicators varied substantially across subgroups of socio‐demographic characteristics. Figure 1 summarises the co‐occurrence of preconception indicators, with additional numerical data shown in Tables S3–S6.

Indicators related to wider determinants of health were highly correlated. For example, women aged under 20 were nearly three times more likely to live in the most deprived areas compared with women aged over 30, while women aged 40 and over were three times less likely to be from an ethnic minority background than were women aged under 20 (Table S3). Women from a black or other ethnic background were twice as likely to live in the most deprived areas compared with women of white ethnicity (Table S4). Compared with women living in the least deprived areas, women living in the most deprived areas were 3.5 times more likely to indicate the presence of complex social factors and 5 times more likely to be unemployed and seeking work (Table S5). Differences in previous pregnancy across socio‐demographic subgroups were smaller (Table S6).

Indicators related to reproductive health, health behaviours and weight, and known pre‐existing health conditions also varied across subgroups of socio‐demographic characteristics. For example, younger women were less likely to have a previous obstetric complication and pre‐existing physical health condition but were also less likely to take folic acid supplementation before pregnancy and more likely to smoke around the time of conception and have a pre‐existing mental health condition. Compared with white women, women from a black ethnic background were more likely to have previously had gestational diabetes and a caesarean section, not to take folic acid supplementation before pregnancy and have obesity, but were less likely to smoke around the time of conception and have a pre‐existing mental health condition. The prevalence of all indicator measures was higher among women living in the most deprived than in the least deprived areas, except for previous caesarean section, previous pregnancy loss, pre‐existing physical health condition and family history of an inherited condition. Compared with women with no previous pregnancy (resulting in live birth and/or pregnancy loss), women who had previously been pregnant were more likely to be living with obesity or a pre‐existing physical health condition (Tables S3–S6).

### 3.2 Priority indicators

In the ranking exercise applied to 15 indicators, the mean score ranged from 6.7 (not taking folic acid supplementation before pregnancy, scored in the top ten by 91% of respondents) to 0.3 (previous caesarean section, scored in top ten by <1%) (Table S7). Indicators that ranked in the top ten comprised not taking folic acid supplementation before pregnancy (unadjusted overall prevalence: 72.7%), obesity (22.3%), complex social factors (12.9%), living in the most deprived areas (14.2%), smoking around conception (19.5%), overweight (28.0%), pre‐existing mental health condition (9.3%), pre‐existing physical health condition (19.1%), previous pregnancy loss (38.9%) and previous obstetric complication (24.5%).

Prevalence estimates for the ten prioritised indicator measures, unadjusted and adjusted for other socio‐demographic characteristics, are presented in Figures S1–S4 and Tables S8–S11 for women with complete data on all relevant variables. The differences in the prevalence of indicators across socio‐demographic characteristics remained present and the distribution was largely similar after adjustment.

### 3.3 Data quality

The proportion of women with missing data on indicators, overall and across subgroups, is shown in Tables S12–S15. There were no missing data for area‐based level of deprivation or for indicators related to obstetric history, pre‐existing physical and mental health conditions, and family history. These latter indicators were only recorded if known as ‘yes’; all other records were missing and assumed to be ‘no’. The proportion of missing data was low for smoking (7.4% missing) and ranged between 15% and 25% for other indicators, except for employment (27.7%) and support status (31.1%).

There were no major differences in the proportions of missing data across subgroups of socio‐demographic characteristics. However, younger women were more likely to have missing data on employment, previous pregnancy loss and weight status. Women from a white ethnic background had a higher proportion of missing data on all indicators, except for folic acid supplementation. Women in the least deprived areas had lower proportion of missing data on support status, and on data not stated (asked but declined to provide a response) for folic acid supplementation (Tables S12–S15).

## 4 DISCUSSION

### 4.1 Main findings

This report card presents the first national‐level overview of the state of preconception health among women in England using data routinely collected by maternity services. Among all pregnant women who attend a first antenatal appointment in England, 9 in 10 have at least one indicator that presents a risk for mother and baby. Priority adverse indicators are common, with for example nearly three‐quarters of women in England not taking a folic acid supplement before pregnancy, half living with overweight or obesity, and about a quarter entering pregnancy with a previous obstetric complication or a pre‐existing physical and/or mental health condition. Strong and independent socio‐demographic inequalities in these preconception indicators were observed. This national picture of preconception health improves our understanding of the population's preconception needs and identifies a need to improve and reduce inequalities for better population health outcomes. It forms the foundation for future report cards and ongoing national surveillance.

### 4.2 Interpretation

The prevalence and clustering of selected preconception indicators have been described at a national level in few previous international studies and reports. Routinely collected maternity data from Northern Ireland have shown that 22.5% of women entered pregnancy with obesity in 2017, an increase from 18.4% in 2010.^22^ Women living with obesity were more likely to be older, parous, unemployed, live in the most deprived areas and report an unplanned pregnancy.^22^ Similarly, in Australia, 21.1% of women had obesity in early pregnancy in 2019.^23^ The latest Australian Mothers and Babies report further shows that 25% of mothers were aged ≥35 and 1.2% <20 years at the birth of their baby, 31.9% had previously given birth by caesarean section, 0.9% had pre‐existing diabetes and 0.6% had pre‐existing hypertension.^23^ The prevalence of smoking among women of reproductive age in 2018/2019 was approximately 20% in Northern Ireland,^24^ 11% in Australia^25^ and 18% in Scotland.^26^ These findings are comparable with prevalence estimates we report in England for obesity and pre‐existing conditions, while the prevalence was lower for advanced maternal age and previous caesarean section compared with Australia, higher for smoking compared with Northern Ireland, Australia and Scotland, and higher for teenage pregnancies compared with Australia. Although teenage pregnancies more than halved during the 10‐year implementation period of the Teenage Pregnancy Strategy for England (1998–2014), the data presented here indicate that youth remains an important marker of social disadvantage and higher risk pregnancies.

In the USA, comprehensive monitoring of core preconception health and care indicators has been part of public health surveillance by the Centers for Disease Control and Prevention (CDC) for more than a decade. Reports based on state‐ and population‐based survey data collected in 2004 and 2009 among women who recently delivered a live‐born infant have shown no significant changes in the overall prevalence for seven of ten indicators that were defined consistently. These included indicators related to contraceptive use, medical conditions, physical abuse, social and emotional support, and receipt of a postpartum check‐up.^27^, ^28^ The prevalence worsened during this time for three indicators; smoking (23.2–25.1%), any alcohol use (50.1–54.2%) 3 months before pregnancy and previous preterm delivery (11.9–14.4%). Stark differences were observed in the prevalence of these indicators by maternal age and ethnicity in the USA, with patterns of results similar to those of our study for most comparable indicators. For example, based on data from the USA and UK, a higher prevalence was observed of not using folic acid supplementation before pregnancy among younger women and women from ethnic minority backgrounds, smoking and mental health conditions among younger women and White women, and obesity among older women and women from a black ethnic background.^27^, ^28^ Differences across countries and regions in availability of preconception care, policy priorities and in sources and methods of routine data collection mean that priority indicators are likely to differ internationally. International collaboration could facilitate alignment of indicator definitions where possible, to allow benchmarking and accountability.

### 4.3 Data strengths and limitations

This first report card fills an important gap in preconception health surveillance in England. Building on the two indicators already included in existing surveillance by OHID (folic acid supplementation before pregnancy and obesity in early pregnancy),^16^ we identified eight additional indicators that could be prioritised for ongoing surveillance. Moreover, we report on a total of 32 indicator measures to provide a comprehensive baseline picture of preconception health in England. The stratification of all indicators by key socio‐demographic characteristics revealed mixed patterns of inequalities, demonstrating for example differences across area‐based deprivation level for obesity but not overweight, and for pre‐existing mental health but not physical health conditions. The MSDS is a unique national data source which records annual preconception indicator data (including wider determinants of health) for all pregnant women with a booking appointment in England.

Limitations related to the use of the MSDS include data quality and lack of data on additional key indicators; Box 1 recommends that these be addressed to improve ongoing national surveillance. A substantial proportion of data were missing for most indicators reported in the MSDS (>15%), with some differences by age, ethnicity, deprivation and previous pregnancy which may have biased prevalence estimates. There were no missing data for mandatory items where failure to submit data results in rejection of the submission, including indicators related to obstetric history, pre‐existing physical and mental health conditions, and family history. Data items related to all other indicators were required (i.e. relevant to clinical practice guidelines but health professionals can continue to enter data if not recorded; missing data 7.4–31.1%), except complex social factors, which is an optional data item (i.e. to be submitted at health professional's discretion; missing data 19.2%). While completion rates are expected to improve as a result of incentivising NHS Trusts, improving data feedback and IT systems, reasons for missing data could be explored at the Trust and healthcare professional levels to improve data quality. Data recording and collection could also be improved, for example, by disaggregating indicators such as complex social factors (aged <20, experienced domestic abuse, recent migrant/asylum seeker/refugee, difficulty reading/speaking English, substance misuse) and previous caesarean section (based on underlying reason that may present a risk for a future pregnancy), and by objectively measuring indicators in all Trusts such as height, weight, smoking and substance use to avoid reliance on self‐reported data. Data on obstetric history, pre‐existing physical and mental health conditions and family history could be improved by adding options for ‘no’ and ‘unknown’, or by obtaining data from previous (linked) health records, to reduce misclassification bias and potential over‐ and underreporting associated with the current self‐reporting of ‘yes’, and ‘no’ assumed when data are missing. Additional preconception indicators that are highly relevant to inform a woman's maternity care pathway could be considered for inclusion in the national MSDS; an example is the London Measure of Unplanned Pregnancy (LMUP), for which evaluation of integration in antenatal care is underway.

BOX 1 **Suggested actions and next steps towards comprehensive ongoing national surveillance in England.**

- Annual reporting of (priority) indicators through a Preconception Health Profile within the government Office for Health Improvement and Disparities' existing surveillance platform, starting with indicators recorded in the national Maternity Services Dataset
- Optimising the utility of maternity services data for surveillance purposes, by improving data quality (e.g. reducing the proportion of missing data and the reliance on self‐reported data), and integrating additional key indicators (e.g. London Measure of Unplanned Pregnancy, LMUP)
- Exploring the use of additional and potentially better‐quality preconception indicators recorded in other core data sources, such as primary care and community services datasets as well as population‐based surveys
- Co‐development of system‐ and policy‐level indicators, to evaluate national strategies (e.g. the new Women's Health Strategy for England) and policy changes (e.g. the forthcoming introduction of mandatory folic acid fortification)
- Linkage of national core datasets to enable monitoring of indicators recorded across multiple health services (e.g. contraceptive use) and evaluation of the impact of changes in preconception health on reducing adverse outcomes and inequalities for mothers and children
- Ongoing review of (priority) indicators based on changes in routine data collection, available data sources and national priorities

In addition to improving the use of the MSDS for national surveillance, other core data sources could be explored. These might collectively include data on people of all genders, at all stages of their reproductive years, irrespective of pregnancy history and intention. Further indicators (e.g. housing, routine health check‐ups, teratogenic medication use and previous breastfeeding experience) and better‐quality indicators (e.g. an uncontrolled or unreviewed specific medical conditions, rather than presence of conditions as recorded in the current report card) could be obtained, for example through routine primary care and community services data as well as non‐healthcare data.^3^

Data recorded in the MSDS are mostly individual‐level risk factors and further system‐ and policy‐level indicators could be co‐developed; these could, for example, evaluate the inclusion of preconception health in mandatory school curricula. Indicators could also be co‐developed to evaluate goals outlined in relevant policy strategies, including the new Women's Health Strategy for England^29^ and the UK Maternity Disparities Taskforce.^30^ The impact of mandatory folic acid fortification announced in 2021^31^ on, for example, adequate blood folate levels among women of reproductive age could be evaluated, alongside trends in neural tube defects and associated health consequences. Together with individual‐level indicators, these system‐ and policy‐level indicators will provide evidence on (inequalities in) women's receipt of preconception health education, promotion and care and on the impact of policies and interventions.

Additional work is also needed to build stronger evidence on the health and cost benefit of improving preconception health, and to link national core datasets to inform a comprehensive and evidence‐based preconception indicator framework. Annual data are needed on the contribution of changes in preconception indicators to maternal and child health outcomes, and on the return on investment of improved preconception health and reduced inequalities. This would inform evidence‐based and annually reviewed prioritisation of preconception indicators and of interventions and policies that address them. Linkage of core datasets is needed to improve the quality of indicators; for example, linking MSDS data with data from other health services such as primary care, community services, and sexual and reproductive health services will enable comprehensive assessment of preconception indicators recorded across multiple health services such as contraceptive use and fertility treatment.

## 5 CONCLUSION

We provide a first national picture of the current state of preconception health among women in England based on routine maternity services data. Findings indicate that population‐level policies and programmes are needed to optimise the health, behaviours and their wider social, economic and environmental determinants among all women of reproductive age, and that various socio‐demographic subgroups may require targeted interventions. Further development of a comprehensive national surveillance infrastructure that utilises multiple linked routine datasets to monitor a range of individual‐, system‐ and policy‐level indicators could offer opportunities to inform, evaluate and prioritise new and existing policies and programmes. This would support the Women's Health Strategy for England, the UK Maternity Disparities Taskforce and the wider Levelling Up agenda by making better use of data collected from health and care services, gaining a better understanding of the drivers behind disparities in adverse pregnancy outcomes, and measuring the strategy's successes, ultimately improving outcomes for all women and children.

## AUTHOR CONTRIBUTIONS

DAJMS, JS and NAA conceived the study. All authors were involved in the design. DAJMS and NAA conceived the analysis plan. DAJMS analysed the data. HS replicated the analysis and verified the results. DAJMS interpreted the results and wrote the paper, with contributions from all authors. All authors approved the final version for submission.

## FUNDING INFORMATION

DAJMS is supported by the National Institute for Health and Social Care Research (NIHR), Southampton Biomedical Research Centre [IS‐BRC‐1215‐20004]. KMG is supported by the UK Medical Research Council (MC_UU_12011/4), the NIHR (NIHR Senior Investigator [NF‐SI‐0515‐10042] and NIHR Southampton Biomedical Research Centre [IS‐BRC‐1215‐20004]), the European Union (Erasmus+ Programme ImpENSA 598488‐EPP‐1‐2018‐1‐DE‐EPPKA2‐CBHE‐JP) and the British Heart Foundation (RG/15/17/3174). The views expressed are those of the authors and not necessarily those of the NIHR or the Department of Health and Social Care. For the purpose of Open Access, the authors have applied a Creative Commons Attribution (CC BY) licence to any Author Accepted Manuscript version arising from this submission.

## CONFLICT OF INTEREST STATEMENT

KMG has received reimbursement for speaking at conferences sponsored by companies selling nutritional products, and is part of an academic consortium that has received research funding from Abbott Nutrition, Nestec, BenevolentAI Bio Ltd. and Danone. The other authors have no conflicts of interest to disclose. Completed disclosure of interests forms are available to view online as supporting information.

## DETAILS OF ETHICS APPROVAL

Ethical approval for the current study was granted by the University of Southampton Faculty of Medicine Ethics Committee (ID 57993) and the NHS Health Research Authority and Research Ethics Committee (IRAS ID 285601; REC reference 20/WM/0231).

## Supporting information


Appendix S1

Data S1

Data S2

Data S3

Data S4

Data S5

Data S6

Data S7

Data S8

Data S9

## Data Availability

Data used for this study were collected for the national Maternity Services Dataset and are available on request from NHS Digital (https://digital.nhs.uk/services/data‐access‐request‐service‐dars).

## References

[1] Stephenson J , Heslehurst N , Hall J , Schoenaker D , Hutchinson J , Cade JE , et al. Before the beginning: nutrition and lifestyle in the preconception period and its importance for future health. Lancet. 2018;391(10132):1830–41. 29673873 10.1016/S0140-6736(18)30311-8 PMC6075697

[2] Fleming TP , Watkins AJ , Velazquez MA , Mathers JC , Prentice AM , Stephenson J , et al. Origins of lifetime health around the time of conception: causes and consequences. Lancet. 2018;391(10132):1842–52. 29673874 10.1016/S0140-6736(18)30312-X PMC5975952

[3] Schoenaker DA , Stephenson J , Connolly A , Shillaker S , Fishburn S , Barker M , et al. Characterising and monitoring preconception health in England: a review of national population‐level indicators and core data sources. J Dev Orig Health Dis. 2022;13(2):137–50. 34085623 10.1017/S2040174421000258 PMC7612507

[4] Dorney E , Boyle J , Walker R , Hammarberg K , Musgrave L , Schoenaker DA , et al. A systematic review of clinical guidelines for preconception care. Semin Reprod Med. 2022;40(3‐04):157–69. 35576970 10.1055/s-0042-1748190

[5] National Institute for Health and Care Excellence (NICE) . Pre‐conception – advice and management. 2021 [cited 2022 Oct 27]. Available from: https://cks.nice.org.uk/pre‐conception‐advice‐and‐management

[6] Public Health England . Making the case for preconception care. 2018 [cited 2022 Oct 27]. Available from: https://assets.publishing.service.gov.uk/government/uploads/system/uploads/attachment_data/file/729018/Making_the_case_for_preconception_care.pdf

[7] World Health Organization (WHO) . Preconception care. Mazimizing the gains for maternal and child health. Policy brief. 2012 [cited 2022 Oct 27]. Available from: https://www.who.int/maternal_child_adolescent/documents/preconception_care_policy_brief.pdf

[8] Centers for Disease Control and Prevention (CDC) . Recommendations to imrpove preconception health and care – United States. A report of the CDC/ATSDR Preconception Care Work Group and the Select Panel of Preconception Care. 2006 [cited 2022 Oct 27]. Available from: https://www.cdc.gov/mmwr/preview/mmwrhtml/rr5506a1.htm

[9] NHS South East Clinical Delivery and Networks . Ready for pregnancy campaign. 2021 [cited 2022 Oct 27]. Available from: https://www.southeastclinicalnetworks.nhs.uk/readyforpregnancy/

[10] Institute of Health Visiting . Sexual and reproductive health resources. 2021 [cited 2022 Oct 27]. Available from: https://ihv.org.uk/for‐health‐visitors/resources/resource‐library‐a‐z/sexual‐and‐reproductive‐health‐resources/

[11] Children's Alliance . The health & wellbeing of children in the early years. 2021 [cited 2022 Oct 27]. Available from: https://childrensalliance.org.uk/2021/10/22/the‐early‐years‐report/

[12] Barker M , Dombrowski SU , Colbourn T , Fall CHD , Kriznik NM , Lawrence WT , et al. Intervention strategies to improve nutrition and health behaviours before conception. Lancet. 2018;391(10132):1853–64. 29673875 10.1016/S0140-6736(18)30313-1 PMC6075694

[13] UK Preconception Partnership [cited 2022 Oct 27]. Available from: http://www.ukpreconceptionpartnership.co.uk/

[14] Stephenson J , Vogel C , Hall J , Hutchinson J , Mann S , Duncan H , et al. Preconception health in England: a proposal for annual reporting with core metrics. Lancet. 2019;393(10187):2262–71. 31162084 10.1016/S0140-6736(19)30954-7

[15] Office for Health Improvement and Disparities (OHID) . Public health profiles. 2022 [cited 2022 Oct 27]. Available from: https://fingertips.phe.org.uk/

[16] Office for Health Improvement and Disparities (OHID) . Public health profiles – child and maternal health. 2022 [cited 2022 Oct 27]. Available from: https://fingertips.phe.org.uk/profile/child‐health‐profiles

[17] NHS Digital . Maternity Services Dataset. [cited 2022 Oct 27]. Available from: https://digital.nhs.uk/Maternity‐Services‐Data‐Set

[18] NHS Digital . Maternity Services Dataset v1.5 archived guidance documents. [cited 2022 Oct 27]. Available from: https://digital.nhs.uk/data‐and‐information/data‐collections‐and‐data‐sets/data‐sets/maternity‐services‐data‐set/archived‐guidance‐documents

[19] National Institute for Health and Care Excellence (NICE) . Antenatal care. NICE guideline [NG201]. 2021 [cited 2022 Oct 27]. Available from: https://www.nice.org.uk/guidance/ng201

[20] Inskip H , Crozier S , Baird J , Hammond J , Robinson S , Cooper C , et al. Measured weight in early pregnancy is a valid method for estimating pre‐pregnancy weight. J Dev Orig Health Dis. 2021;12(4):561–9. 33046167 10.1017/S2040174420000926

[21] Inskip HM , Crozier SR , Godfrey KM , Borland SE , Cooper C , Robinson SM . Women's compliance with nutrition and lifestyle recommendations before pregnancy: general population cohort study. BMJ. 2009;338:b481. 19213768 10.1136/bmj.b481 PMC2643441

[22] Kent L , Cardwell C , Young I , Eastwood KA . Trends in maternal body mass index in Northern Ireland: a cross‐sectional and longitudinal study. Fam Med Community Health. 2021;9(4):e001310. 34949675 10.1136/fmch-2021-001310 PMC8710425

[23] Alexander J , Cotter D , Parayiwa C , Rek J , Sedgwick K , Welch M . Australia's mothers and babies 2019 – web report. Sydney: AIHW National Perinatal Statistics Unit; 2021 [cited 2022 Oct 27]. Available from https://www.aihw.gov.au/reports/mothers‐babies/australias‐mothers‐babies/contents/about

[24] Department of Health . Health survey Northern Ireland: smoking trends. Last updated April 2022 [cited 2022 Oct 27]. Available from: https://www.health‐ni.gov.uk/publications/health‐survey‐northern‐ireland‐smoking‐trends

[25] Australian Institute of Health and Welfare . Alcohol, tobacco & other drugs in Australia. Last updated August 2022 [cited 2022 Oct 27]. Available from: https://www.aihw.gov.au/reports/alcohol/alcohol‐tobacco‐other‐drugs‐australia/data‐tables

[26] Public Health Information for Scotland . Tobacco use: adult smoking in Scotland. Last updated February 2022 [cited 2022 Oct 27]. Available from: https://www.scotpho.org.uk/behaviour/tobacco‐use/data/adult‐smoking‐in‐scotland/

[27] D'Angelo D , Williams L , Morrow B , Cox S , Harris N , Harrison L , et al. Preconception and interconception health status of women who recently gave birth to a live‐born infant–Pregnancy Risk Assessment Monitoring System (PRAMS), United States, 26 reporting areas, 2004. MMWR Surveill Summ. 2007;56(10):1–35. 18075488

[28] Robbins CL , Zapata LB , Farr SL , Kroelinger CD , Morrow B , Ahluwalia I , et al. Core state preconception health indicators – pregnancy risk assessment monitoring system and behavioral risk factor surveillance system, 2009. MMWR Surveill Summ. 2014;63(3):1–62. 24759729

[29] UK Government Department of Health and Social Care . Policy paper: our vision for the women's health strategy for England. 2021 [cited 2022 Oct 27]. Available from: https://www.gov.uk/government/publications/our‐vision‐for‐the‐womens‐health‐strategy‐for‐england

[30] UK Government Department of Health and Social Care . New taskforce to level‐up maternity care and tackle disparities. 2022 [cited 2022 Oct 27]. Available from: https://www.gov.uk/government/news/new‐taskforce‐to‐level‐up‐maternity‐care‐and‐tackle‐disparities

[31] UK Government Department of Health and Social Care . Folic acid added to flour to prevent spinal conditions in babies. 2021 [cited 2022 Oct 27]. Available from: https://www.gov.uk/government/news/folic‐acid‐added‐to‐flour‐to‐prevent‐spinal‐conditions‐in‐babies

