# Supplementary material for: Women's preconception health in England: a report card based on cross‐sectional analysis of national maternity services data from 2018/2019
Source: BJOG. 2023 Mar 21;130(10):1187–95. doi: 10.1111/1471-0528.17436 (PMC10952348; doi:10.1111/1471-0528.17436)
Supplement: Supplementary file 1 — Appendix S1 [file BJO-130-1187-s001.docx]

**SUPPORTING INFORMATION**

**Table of contents**

|  | **Page** |
| --- | --- |
| **Figure S1.** Unadjusted (blue) and adjusted (grey) prevalence with 95% confidence intervals of 10 prioritised preconception indicators by maternal age | 2 |
| **Figure S2.** Unadjusted (blue) and adjusted (grey) prevalence with 95% confidence intervals of 10 prioritised preconception indicators by ethnicity | 3 |
| **Figure S3.** Unadjusted (blue) and adjusted (grey) prevalence with 95% confidence intervals of 10 prioritised preconception indicators by area-based level of deprivation | 4 |
| **Figure S4.** Unadjusted (blue) and adjusted (grey) prevalence with 95% confidence intervals of 10 prioritised preconception indicators by previous pregnancy | 5 |
| **Table S1. Overview and definitions of preconception indicators recorded in the national Maternity Services Dataset** | 6 |
| **Table S2.** **List of preconception indicators not recorded in the national Maternity Services Dataset** | 12 |
| **Table S3.** Unadjusted prevalence of preconception indicators by maternal age, N = 652,871 | 13 |
| **Table S4.** Unadjusted prevalence of preconception indicators by ethnicity, N = 549,552 | 16 |
| **Table S5.** Unadjusted prevalence of preconception indicators by area-based level of deprivation, N = 652,880 | 19 |
| **Table S6.** Unadjusted prevalence of preconception indicators by previous pregnancy, N = 524,572 | 22 |
| **Table S7.** Ranking of preconception indicators by members of the UK Preconception Partnership, N = 27 | 24 |
| **Table S8.** Unadjusted and adjusted prevalence of 10 prioritised preconception indicators by maternal age | 25 |
| **Table S9.** Unadjusted and adjusted prevalence of 10 prioritised preconception indicators by ethnicity | 28 |
| **Table S10.** Unadjusted and adjusted prevalence of 10 prioritised preconception indicators by area-based level of deprivation | 31 |
| **Table S11.** Unadjusted and adjusted prevalence of 10 prioritised preconception indicators by previous pregnancy | 33 |
| **Table S12.** Proportions of missing data of preconception indicators by maternal age | 35 |
| **Table S13.** Proportions of missing data of preconception indicators by ethnicity | 36 |
| **Table S14.** Proportions of missing data of preconception indicators by area-based level of deprivation | 37 |
| **Table S15.** Proportions of missing data of preconception indicators by previous pregnancy | 38 |
| References | 39 |

**
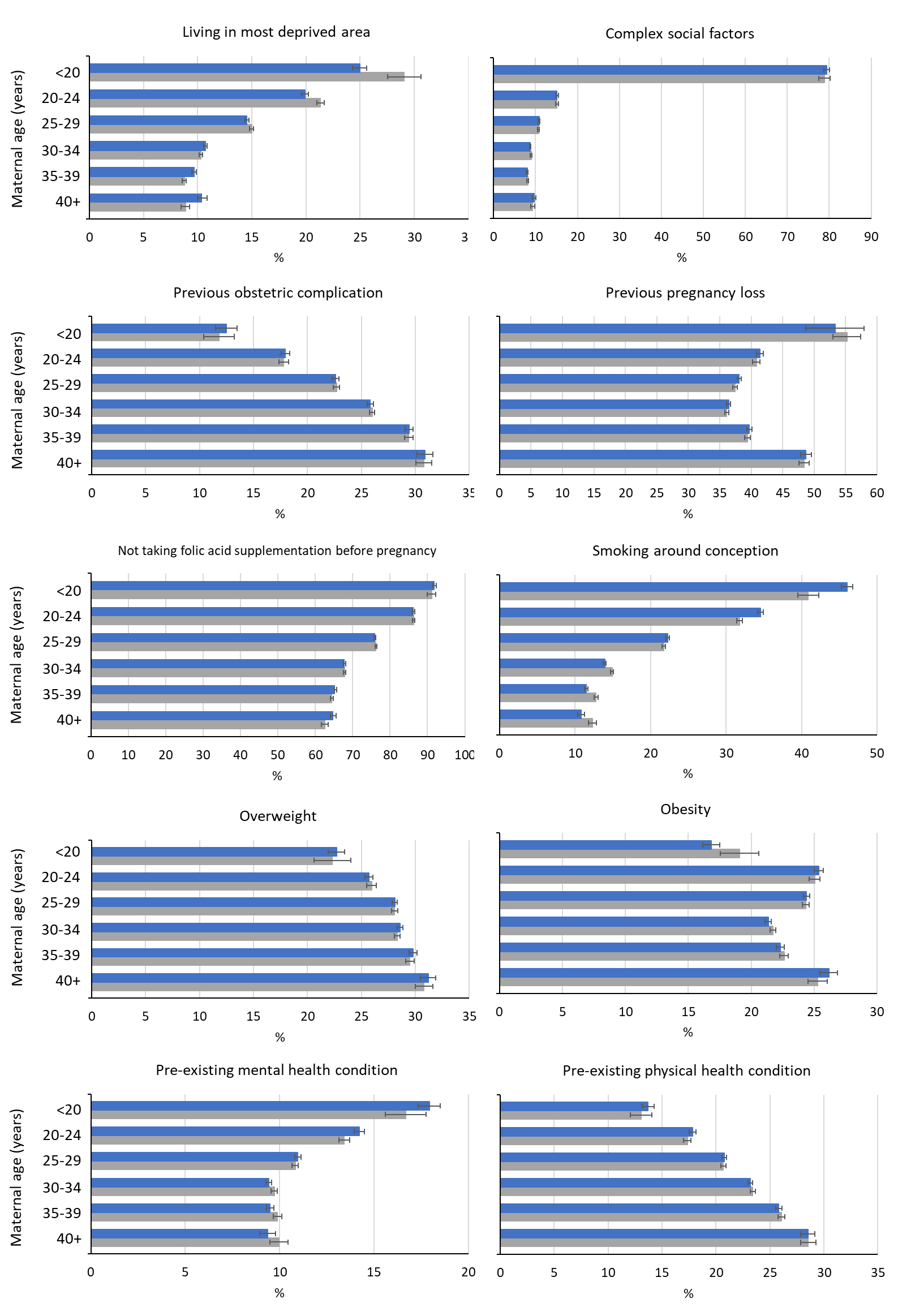
**

**Figure S1.** Unadjusted (blue) and adjusted (grey) prevalence with 95% confidence intervals of 10 prioritised preconception indicators by maternal age. Adjusted for ethnicity, area-based level of deprivation and previous pregnancy.


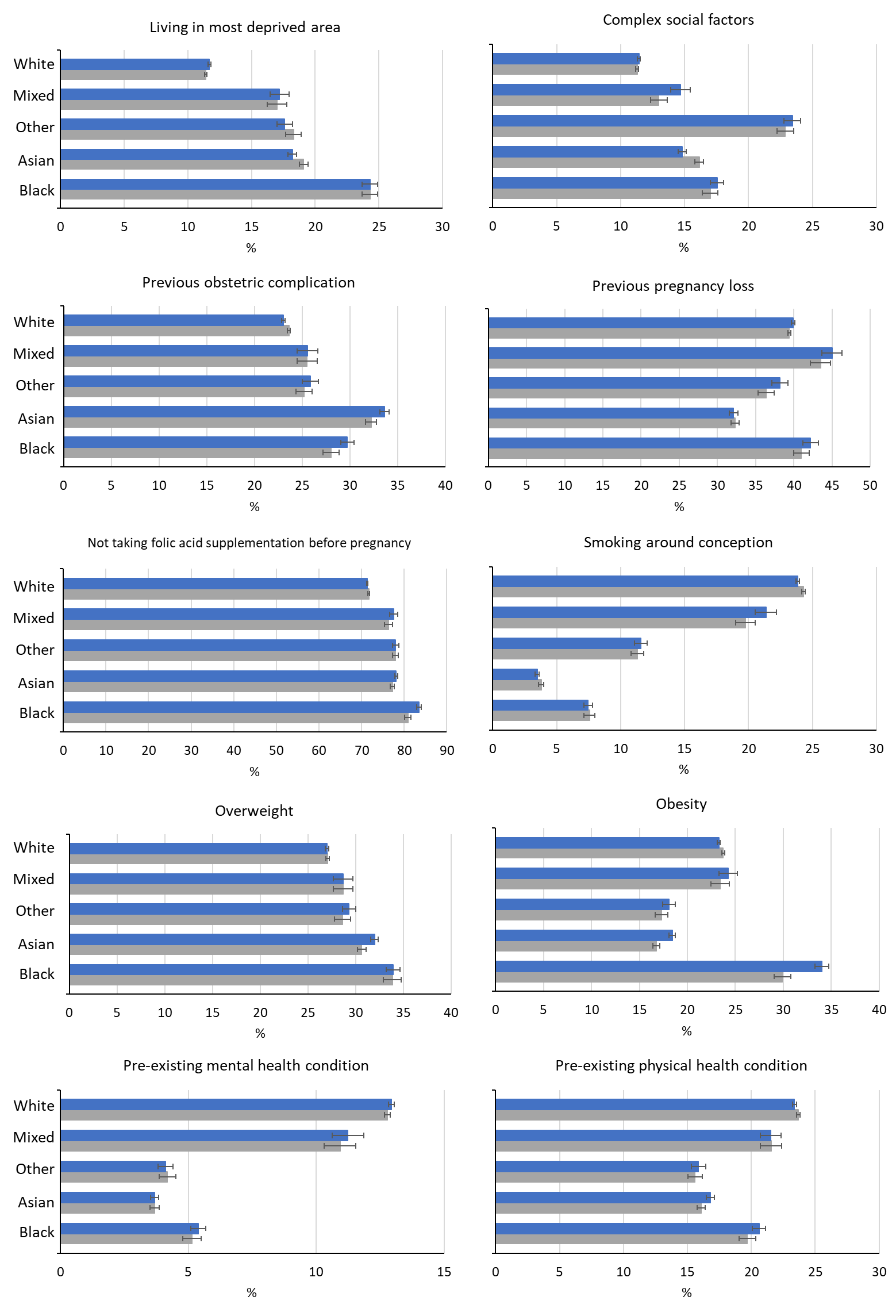


**Figure S2.** Unadjusted (blue) and adjusted (grey) prevalence with 95% confidence intervals of 10 prioritised preconception indicators by ethnicity. Adjusted for maternal age, area-based level of deprivation and previous pregnancy.


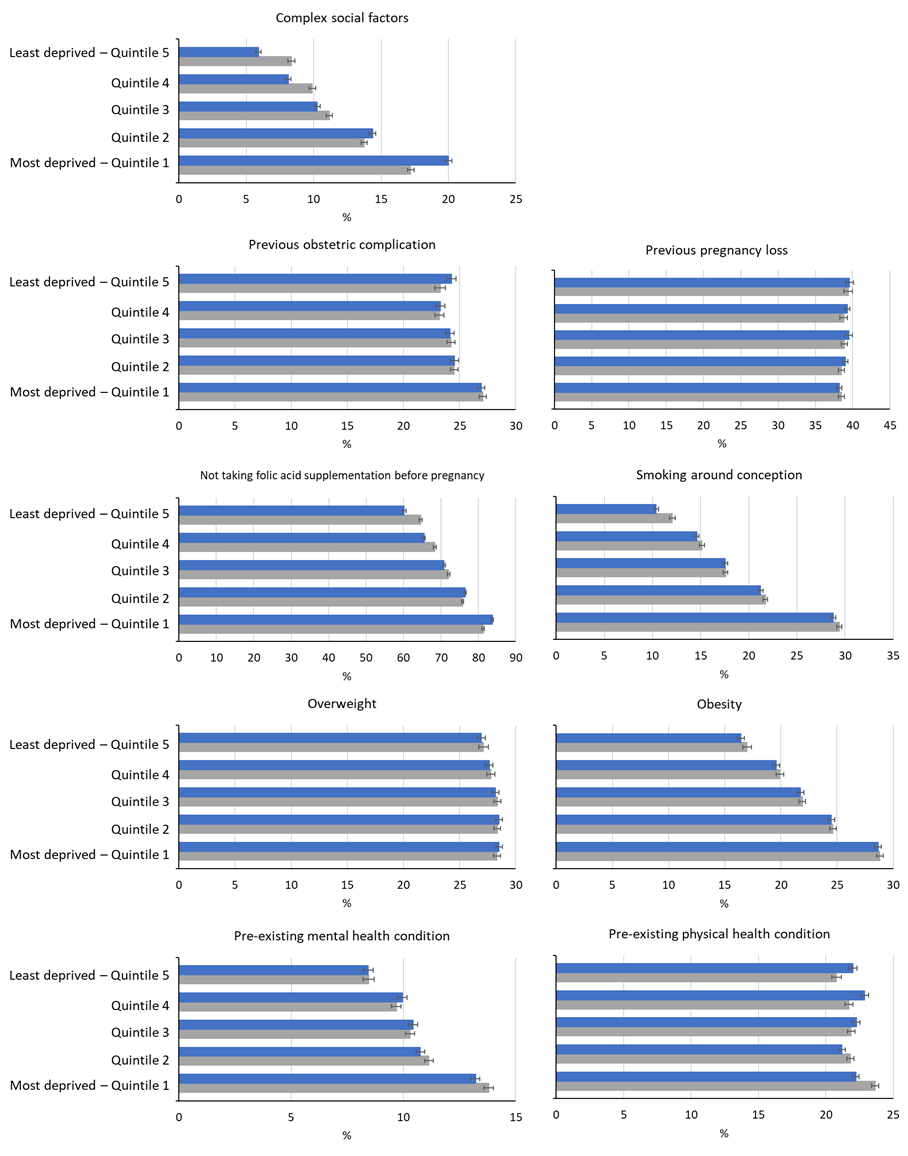


**Figure S3.** Unadjusted (blue) and adjusted (grey) prevalence with 95% confidence intervals of 10 prioritised preconception indicators by area-based level of deprivation. Adjusted for maternal age, ethnicity and previous pregnancy.

**
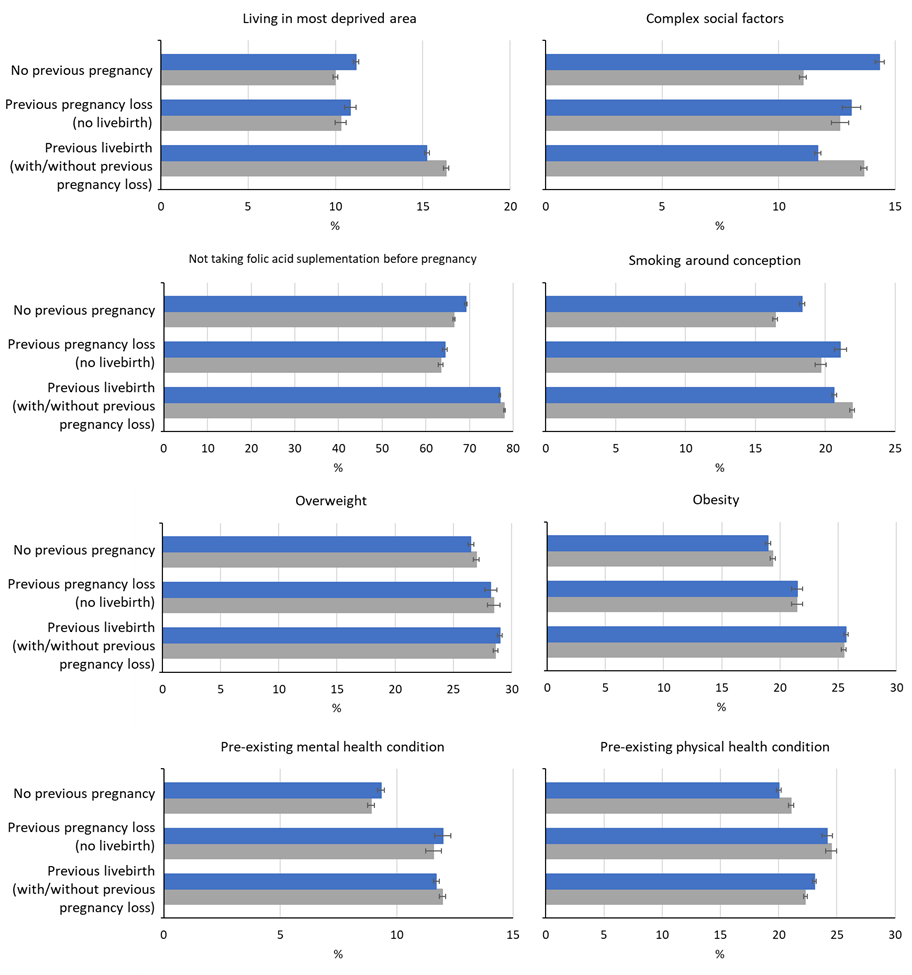
**

**Figure S4**. Unadjusted (blue) and adjusted (grey) prevalence with 95% confidence intervals of 10 prioritised preconception indicators by previous pregnancy. Adjusted for maternal age, ethnicity and area-based level of deprivation.

**Table S1. Overview and definitions of preconception indicators recorded in the national Maternity Services Dataset**

| **Domain** | **Indicator** | **Proposed measure^1^** | **Maternity Services Dataset measure^2^** | **Categories** |
| --- | --- | --- | --- | --- |
| Wider determinants of health | Ethnicity | Percentage of women from a minority ethnic group | As proposed  Data requirement: required | **Yes** (***Mixed*** (White and Black Caribbean, White and Black African, White Asian, Other Mixed background); ***Asian*** (Indian, Pakistani, Bangladeshi, Other Asian background; Chinese); ***Black*** (Caribbean; African; Other Black background); ***Other*** (any other ethnic group))  **No** (***White*** (White British; White Irish; Other White background)) |
|  | Employment | Percentage of women who are unemployed | Percentage of women who are unemployed and seeking work  Data requirement: required | **Yes** (***Unemployed and seeking work*** (Unemployed and seeking work)  **No** (***Employed or not actively seeking work***) (Employed; Students not working or actively seeking work; Long-term sick or disabled, receiving Incapacity Benefit, Income Support or both; Homemaker not working or actively seeking work; Unpaid voluntary work and not working or actively seeking work; Retired) |
|  | Deprivation | Percentage of women living in the most deprived area | As proposed  (based on postcode and the index of multiple deprivation (IMD) 2015)  Data requirement: required | **Yes** (Decile 1)  **No** (Deciles 2 to 9) |
|  | Complex social factors | Percentage of pregnant women who are aged under 20, experience domestic abuse, are recent migrants, asylum seekers or refugees, have difficulty reading or speaking English, or misuse substances (alcohol and/or drugs) | As proposed  Data requirement: optional | **Yes**  **No** |
|  | Migrant health factors | Percentage of immigrant and refugee women | Percentage of women who do not have English as their first language  Data requirement: required | **Yes**  **No** |
| Emotional and social health and support | Support network / social support | Percentage of pregnant women without adequate support available during and after pregnancy (from partner, family and friends) | As proposed  (whether or not the pregnant person feels they are supported in pregnancy and looking after a baby, from partner, family or friends)  Data requirement: required | **Yes**  **No** |
| Reproductive health and family planning | Maternal age | Percentage of pregnant women who are aged 35 years or older | As proposed  (age at booking appointment)  Data requirement: required | **Yes**  **No** |
|  |  | Percentage of pregnant women who are aged below 20 years (teenage pregnancy) | As proposed  Data requirement: required | **Yes**  **No** |
|  | Obstetric history | Percentage of women [who have previously been pregnant] with a history of adverse pregnancy outcomes | As proposed  (based on known diagnosis from a previous pregnancy presenting a risk or complicating factor for this pregnancy)  Data requirement: mandatory | **No** (none)  **Yes** (any of 18 recorded diagnosis) |
|  |  | Percentage of women [who have previously been pregnant] with a history of pre-eclampsia | Percentage of women [who have previously been pregnant] with a history of severe pre-eclampsia requiring pre-term birth; gestational proteinuria; eclampsia; and/or haemolytic anaemia, Elevated Liver enzymes and Low Platelet count (HELLP)  Data requirement: mandatory | **Yes**  **No** |
|  |  | Percentage of women [who have previously been pregnant] with a history of gestational hypertension | As proposed  Data requirement: mandatory | **Yes**  **No** |
|  |  | Percentage of women [who have previously been pregnant] with a history of gestational diabetes | As proposed  Data requirement: mandatory | **Yes**  **No** |
|  |  | Percentage of women [who have previously been pregnant] with a previous caesarean delivery | As proposed  Data requirement: required | **Yes**  **No** |
|  |  | Percentage of women [who have previously been pregnant] with a previous foetal death, miscarriage or stillbirth | Percentage of women [who have previously been pregnant] with a history of stillbirth(s) (gestation at birth >= 24 weeks + 0 days), termination(s) and/or miscarriage(s) (<=23 weeks + 6 days)  Data requirement: required | **Yes**  **No** |
| Health behaviours and weight | Folic acid supplementation | Percentage of pregnant women who did not take a folic acid supplement prior to pregnancy | As proposed  Data requirement: required | **Yes** (Has been taking prior to becoming pregnant)  **No** (Started taking once pregnancy confirmed; Not taking folic acid supplement) |
|  | Tobacco use | Percentage of women who currently smoke | Percentage of women who smoke around the time of conception  Data requirement: required | **Yes** (Current smoker, Ex-smoker - Stopped after conception)  **No** (Ex-smoker - Stopped between conception and 12 months before conception, Ex-smoker - Stopped more than 12 months before conception; Non-smoker - history unknown, Never smoked) |
|  |  | Percentage of pregnant women who smoke and did not stop smoking prior to pregnancy | Percentage of pregnant women who smoked 12 months before pregnancy and did not stop smoking prior to pregnancy  Data requirement: required | **Yes** (Current smoker, Ex-smoker - Stopped after conception)  **No** (Ex-smoker - Stopped between conception and 12 months before conception)  Excluded: Ex-smoker - Stopped more than 12 months before conception; Non-smoker - history unknown, Never smoked |
|  | Weight | Percentage of women in the underweight BMI category (<18.5 kg/m^2^) | As proposed  (weight data were not included if women did not have valid data on gestational age, if gestational age was ≥14 weeks, and if BMI was <13 kg/m^2^ or >80 kg/m^2^, in line with data recorded by the UK Government Office for Health Improvement and Disparities)  Data requirement: required | **Yes** (BMI <18.5 kg/m^2^)  **No** (BMI ≥18.5 kg/m^2^) |
|  |  | Percentage of women in the overweight BMI category (25.0-29.9 kg/m^2^) | As proposed  Data requirement: required | **Yes** (BMI 25.0-29.9 kg/m^2^)  **No** (BMI <25.0 or ≥30 kg/m^2^) |
|  |  | Percentage of women in the obese BMI category (≥30.0 kg/m^2^) | As proposed  Data requirement: required | **Yes** (BMI (≥30.0 kg/m^2^)  **No** (BMI <30 kg/m^2^) |
| Mental health conditions | Mental health condition | Percentage of women with a (uncontrolled or unreviewed) common mental health condition (including depression and anxiety) | Percentage of women with a known mental health condition  Data requirement: mandatory | **Yes**  **No** |
| Physical health conditions | Physical health condition | Percentage of women with a (uncontrolled or unreviewed) physical health condition | Percentage of women with at least one known physical health condition  (based on known past medical history; the diagnosis or type of diagnosis presenting a risk or complicating factor for this pregnancy)  Data requirement: mandatory | **No** (none)  **Yes** (any of 17 recorded diagnosis) |
|  | Health condition | Percentage of women with a (uncontrolled or unreviewed) health condition | Percentage of women with one or more known physical and/or mental health condition(s)  Data requirement: mandatory | **No** (none)  **Yes** (any of 18 recorded diagnosis) |
|  | Diabetes mellitus | Percentage of women with (uncontrolled or unreviewed) type 1 or type 2 diabetes | Percentage of women with known diabetes  Data requirement: mandatory | **Yes**  **No** |
|  | Chronic hypertension | Percentage of women with (uncontrolled or unreviewed) chronic hypertension | Percentage of women with known hypertension  Data requirement: mandatory | **Yes**  **No** |
|  | Cardiovascular disease | Percentage of women with (uncontrolled or unreviewed) cardiovascular disease | Percentage of women with known cardiac disease  Data requirement: mandatory | **Yes**  **No** |
|  | Previous thromboembolism | Percentage of women with previous deep vein thrombosis or pulmonary embolism | Percentage of women with a known thromboembolic condition  Data requirement: mandatory | **Yes**  **No** |
|  | Renal disease | Percentage of women with (uncontrolled or unreviewed) renal disease | Percentage of women with known renal disease  Data requirement: mandatory | **Yes**  **No** |
|  | Hepatitis B | Percentage of women with hepatitis B | Percentage of women with known hepatitis B  Data requirement: mandatory | **Yes**  **No** |
|  | Cancer | Percentage of women with (prior) cancer (treatment) | Percentage of women with known cancer  Data requirement: mandatory | **Yes**  **No** |
| Genetic risk | Personal or family history of a generic condition | Percentage of women with a personal or family history of an inherited genetic condition | Percentage of women with a known family history of an inherited condition  Data requirement: mandatory | **Yes**  **No** |
|  | Family history of diabetes | Percentage of women with a family history of diabetes | As proposed  Data requirement: mandatory | **Yes**  **No** |

^1^ Proposed preconception indicator measures based on Schoenaker et al, 2022 (1)

^2^ Data requirement options: 1) Mandatory: these data items must be reported locally. Failure to submit these items will result in the rejection of the submission; 2) Required: these data items are relevant to clinical practice guidelines and should be reported where they apply. Failure to submit these items will not result in the rejection of the submission but may affect the derivation of national indicators or national analysis; 3) Optional: these data items may be submitted on an optional basis at the submitters discretion (2).

**Table S2.** **List of preconception indicators not recorded in the national Maternity Services Dataset^1^**

| Education |
| --- |
| Financial security |
| Housing |
| Adverse childhood experiences |
| Routine general practitioner (GP) check-up |
| Routine dental appointment |
| Domestic abuse |
| Pregnancy intention |
| Paternal age |
| Interpregnancy interval^2^ |
| Previous breastfeeding experiences |
| Fertility problems |
| Contraception |
| Assisted reproductive technology |
| Vitamin supplementation (other than focus acid) |
| Vitamin deficiency |
| Dietary intake |
| Physical activity |
| Eating disorder |
| Second-hand smoke exposure |
| Alcohol consumption^3^ |
| Substance use^3^ |
| Toxic or hazardous substances or exposures |
| Cervical screening |
| Immunisation |
| Sexually transmitted diseases |
| Previous antenatal or postnatal mental illness |
| Severe mental health condition |
| Epilepsy |
| Polycystic ovary syndrome |
| Endometriosis |
| Thyroid disease |
| Asthma |
| Rheumatological conditions |
| Inflammatory bowel disease |
| Sickle-cell disease or thalassaemia |
| Lupus |
| Phenylketonuria (PKU) |
| Developmental disability |
| Female genital mutilation |
| Pelvic floor dysfunction |
| Medication – overall use |
| Medication – not recommended when planning pregnancy |
| Previous pregnancy affected by an inherited genetic disorder |

^1^ Proposed preconception indicators based on Schoenaker et al, 2022 (1)

^2^ Interpregnancy interval may be calculated when multiple years of MSDS data are available. The current report card is based on one year of MSDS data.

^3^ Alcohol consumption and substance use were reported, but not included in the current report card due to data quality issues.

**Table S3.** Unadjusted prevalence of preconception indicators by maternal age, N = 652,871

|  | **Maternal age (years)** | | | | | |
| --- | --- | --- | --- | --- | --- | --- |
| **Preconception indicator^1^** | **<20** | **20-24** | **25-29** | **30-34** | **35-39** | **40+** |
|  | 24,675 (3.8) | 97,178 (14.9) | 184,186 (28.2) | 207,171 (31.7) | 113,458 (17.4) | 26,203 (4.0) |
|  | n  % (95% CI) | n  % (95% CI) | n  % (95% CI) | n  % (95% CI) | n  % (95% CI) | n  % (95% CI) |
| ***Wider determinants of health*** |  |  |  |  |  |  |
| Ethnic minority (n = 549,546) | 2,316 | 14,466 | 33,645 | 42,629 | 25,223 | 6,820 |
|  | 11.1 (10.7, 11.5) | 17.6 (17.3, 17.9) | 21.8 (21.5, 22.0) | 24.6 (24.4, 24.8) | 26.3 (26.0, 26.6) | 30.7 (30.1, 31.3) |
| Unemployed and seeking work (n = 472,175) | 3,724 | 7,695 | 6,995 | 5,081 | 2,655 | 699 |
|  | 22.2 (21.6, 22.9) | 11.2 (11.0, 11.5) | 5.3 (5.2, 5.4) | 3.3 (3.3, 3.4) | 3.2 (3.1, 3.3) | 3.6 (3.4, 3.9) |
| Living in most deprived area (n = 652,871) | 6,746 | 20,756 | 28,167 | 22,739 | 11,313 | 2,807 |
|  | 27.3 (26.8, 27.9) | 21.4 (21.1, 21.6) | 15.3 (15.1, 15.5) | 11.0 (10.8, 11.1) | 10.0 (9.8, 10.1) | 10.7 (10.3, 11.1) |
| Complex social factors (n = 527,583) | 16,140 | 11,768 | 16,007 | 14,432 | 7,484 | 2,056 |
|  | 80.5 (80.0, 81.0) | 15.3 (15.0, 15.5) | 10.8 (10.7, 11.0) | 8.6 (8.4, 8.7) | 8.0 (7.9, 8.2) | 9.5 (9.2, 9.9) |
| English not as first language (n = 497,636) | 2,820 | 14,408 | 29,015 | 32,754 | 18,301 | 4,690 |
|  | 15.3 (14.8, 15.8) | 19.6 (19.4, 19.9) | 20.8 (20.6, 21.0) | 20.7 (20.5, 20.9) | 20.9 (20.6, 21.1) | 23.0 (22.5, 23.6) |
| ***Emotional and social health and support*** |  |  |  |  |  |  |
| No adequate support available during and after pregnancy (n = 449,877) | 1,199 | 4,180 | 7,214 | 8,038 | 4,773 | 1,186 |
|  | 7.2 (6.8, 7.6) | 6.2 (6.1, 6.4) | 5.7 (5.5, 5.8) | 5.6 (5.5, 5.8) | 6.1 (5.9, 6.3) | 6.6 (6.2, 6.9) |
| ***Reproductive health*** |  |  |  |  |  |  |
| Known previous obstetric complication (n = 329,224) | 581 | 6,970 | 19,225 | 28,276 | 20,454 | 5,188 |
|  | 12.2 (11.3, 13.1) | 17.9 (17.5, 18.3) | 22.4 (22.1, 22.6) | 25.4 (25.1, 25.6) | 28.9 (28.5, 29.2) | 30.2 (29.6, 30.9) |
| Previous pre-eclampsia, HELLP, eclampsia, gestational proteinuria (n = 329,224) | 38 | 382 | 894 | 1,125 | 798 | 232 |
|  | 0.8 (0.6, 1.1) | 1.0 (0.9, 1.1) | 1.0 (1.0, 1.1) | 1.0 (1.0, 1.1) | 1.1 (1.1, 1.2) | 1.4 (1.2, 1.5) |
| Previous gestational hypertension (n = 329,224) | 27 | 464 | 1,334 | 1,734 | 1,283 | 366 |
|  | 0.6 (0.4, 0.8) | 1.2 (1.1, 1.3) | 1.6 (1.5, 1.6) | 1.6 (1.5, 1.6) | 1.8 (1.7, 1.9) | 2.1 (1.9, 2.4) |
| Previous gestational diabetes mellitus (n = 329,224) | 32 | 465 | 1,537 | 2,673 | 2,181 | 618 |
|  | 0.7 (0.5, 0.9) | 1.2 (1.1, 1.3) | 1.8 (1.7, 1.9) | 2.4 (2.3, 2.5) | 3.1 (3.0, 3.2) | 3.6 (3.3, 3.9) |
| Previous caesarean section (n = 306,426) | 391 | 5,351 | 15,514 | 24,725 | 18,939 | 5,070 |
|  | 9.0 (8.2, 9.9) | 14.9 (14.5, 15.3) | 19.4 (19.1, 19.7) | 23.8 (23.5, 24.0) | 28.6 (28.2, 28.9) | 31.6 (30.9, 32.3) |
| Previous pregnancy loss (n = 301,164) | 2,352 | 14,770 | 29,782 | 37,010 | 25,685 | 7,656 |
|  | 52.8 (51.3, 54.2) | 41.3 (40.8, 41.8) | 37.9 (37.6, 38.3) | 36.4 (36.1, 36.7) | 39.6 (39.2, 40.0) | 48.4 (47.6, 49.2) |
| ***Health behaviours and weight*** |  |  |  |  |  |  |
| Not taken folic acid supplementation before pregnancy (n = 488,980) | 16,720 | 62,678 | 10,4715 | 10,4278 | 54,748 | 12,504 |
|  | 91.6 (91.1, 92.0) | 85.8 (85.6, 86.1) | 75.4 (75.2, 75.6) | 67.3 (67.0, 67.5) | 64.9 (64.6, 65.2) | 64.5 (63.8, 65.2) |
| Smoking around conception (n = 604,506) | 10,145 | 30,034 | 36,252 | 26,225 | 12,270 | 2,675 |
|  | 44.9 (44.2, 45.5) | 33.5 (33.2, 33.8) | 21.3 (21.1, 21.5) | 13.6 (13.5, 13.8) | 11.6 (11.4, 11.8) | 11.0 (10.6, 11.4) |
| Smokers who did not quit smoking during year before pregnancy (n = 138,421) | 10,145 | 30,034 | 36,252 | 26,225 | 12,270 | 2,675 |
|  | 90.5 (90.0, 91.1) | 88.0 (87.7, 88.4) | 84.9 (84.6, 85.2) | 81.9 (81.5, 82.4) | 81.0 (80.4, 81.6) | 82.3 (80.9, 83.6) |
| Underweight at booking (BMI <18.5 kg/m^2^) (n = 496,327) | 1,534 | 3,706 | 4,365 | 3,884 | 1,556 | 301 |
|  | 9.2 (8.8, 9.6) | 5.2 (5.0, 5.4) | 3.1 (3.0, 3.2) | 2.4 (2.3, 2.5) | 1.8 (1.7, 1.9) | 1.5 (1.4, 1.7) |
| Overweight at booking (BMI 25 to 29.9 kg/m^2^) (n = 496,327) | 3,787 | 18,354 | 39,084 | 45,510 | 25,887 | 6,150 |
|  | 22.7 (22.1, 23.3) | 25.8 (25.5, 26.1) | 27.8 (27.6, 28.0) | 28.3 (28.1, 28.5) | 29.5 (29.2, 29.8) | 31.4 (30.7, 32.0) |
| Obesity at booking (BMI ≥30 kg/m^2^) (n = 496,327) | 2,807 | 17,687 | 33,207 | 33,023 | 18,969 | 4,933 |
|  | 16.8 (16.3, 17.4) | 24.9 (24.5, 25.2) | 23.6 (23.4, 23.8) | 20.6 (20.4, 20.7) | 21.6 (21.4, 21.9) | 25.2 (24.6, 25.8) |
| ***Known pre-existing health conditions*** |  |  |  |  |  |  |
| At least one health condition (n = 652,871) | 5,400 | 22,008 | 42,721 | 50,591 | 30,482 | 7,635 |
|  | 21.9 (21.4, 22.4) | 22.6 (22.4, 22.9) | 23.2 (23.0, 23.4) | 24.4 (24.2, 24.6) | 26.9 (26.6, 27.1) | 29.1 (28.6, 29.7) |
| Mental health condition (n = 652,871) | 3,618 | 11,684 | 17,286 | 16,713 | 9,472 | 2,198 |
|  | 14.7 (14.2, 15.1) | 12.0 (11.8, 12.2) | 9.4 (9.3, 9.5) | 8.1 (7.9, 8.2) | 8.3 (8.2, 8.5) | 8.4 (8.1, 8.7) |
| Physical health condition (n = 652,871) | 2,749 | 14,648 | 32,951 | 41,839 | 25,877 | 6,641 |
|  | 11.1 (10.8, 11.5) | 15.1 (14.8, 15.3) | 17.9 (17.7, 18.1) | 20.2 (20.0, 20.4) | 22.8 (22.6, 23.1) | 25.3 (24.8, 25.9) |
| Diabetes (n = 652,871) | 122 | 626 | 1,444 | 2,060 | 1,565 | 526 |
|  | 0.5 (0.4, 0.6) | 0.6 (0.6, 0.7) | 0.8 (0.7, 0.8) | 1.0 (1.0, 1.0) | 1.4 (1.3, 1.4) | 2.0 (1.8, 2.2) |
| Hypertension (n = 652,871) | 71 | 605 | 1,537 | 2,145 | 1,701 | 637 |
|  | 0.3 (0.2, 0.4) | 0.6 (0.6, 0.7) | 0.8 (0.8, 0.9) | 1.0 (1.0, 1.1) | 1.5 (1.4, 1.6) | 2.4 (2.3, 2.6) |
| Cardiac disease (n = 652,871) | 173 | 841 | 1,480 | 1,580 | 910 | 200 |
|  | 0.7 (0.6, 0.8) | 0.9 (0.8, 0.9) | 0.8 (0.8, 0.8) | 0.8 (0.7, 0.8) | 0.8 (0.8, 0.9) | 0.8 (0.7, 0.9) |
| Thromboembolic condition (n = 652,871) | 45 | 359 | 959 | 1,335 | 928 | 259 |
|  | 0.2 (0.1, 0.2) | 0.4 (0.3, 0.4) | 0.5 (0.5, 0.6) | 0.6 (0.6, 0.7) | 0.8 (0.8, 0.9) | 1.0 (0.9, 1.1) |
| Renal disease (n = 652,871) | 191 | 888 | 1,552 | 1,519 | 789 | 187 |
|  | 0.8 (0.7, 0.9) | 0.9 (0.9, 1.0) | 0.8 (0.8, 0.9) | 0.7 (0.7, 0.8) | 0.7 (0.6, 0.7) | 0.7 (0.6, 0.8) |
| Hepatitis B (n = 652,871) | 19 | 82 | 250 | 338 | 244 | 65 |
|  | 0.1 (0.0, 0.1) | 0.1 (0.1, 0.1) | 0.1 (0.1, 0.2) | 0.2 (0.1, 0.2) | 0.2 (0.2, 0.2) | 0.2 (0.2, 0.3) |
| Cancer (n = 652,871) | 16 | 69 | 260 | 360 | 288 | 92 |
|  | 0.1 (0.0, 0.1) | 0.1 (0.1, 0.1) | 0.1 (0.1, 0.2) | 0.2 (0.2, 0.2) | 0.3 (0.2, 0.3) | 0.4 (0.3, 0.4) |
| ***Known family history*** |  |  |  |  |  |  |
| Inherited conditions (n = 652,871) | 586 | 2,166 | 3,819 | 4,104 | 2,167 | 481 |
|  | 2.4 (2.2, 2.6) | 2.2 (2.1, 2.3) | 2.1 (2.0, 2.1) | 2.0 (1.9, 2.0) | 1.9 (1.8, 2.0) | 1.8 (1.7, 2.0) |
| Diabetes (n = 652,871) | 4,195 | 19,072 | 37,941 | 43,293 | 24,068 | 5,828 |
|  | 17.0 (16.5, 17.5) | 19.6 (19.4, 19.9) | 20.6 (20.4, 20.8) | 20.9 (20.7, 21.1) | 21.2 (21.0, 21.5) | 22.2 (21.7, 22.7) |

BMI, body mass index; CI, confidence interval; HELLP, Elevated Liver enzymes and Low Platelet count

^1^ Definitions of indicators can be found in Supplementary Table 1.

**Table S4.** Unadjusted prevalence of preconception indicators by ethnicity, N = 549,552

|  | **Ethnicity** | | | | |
| --- | --- | --- | --- | --- | --- |
| **Preconception indicator**^1^ | **White** | **Mixed** | **Asian** | **Black** | **Other** |
|  | 424,453 (77.2) | 11,695 (2.1) | 66,444 (12.1) | 26,172 (4.8) | 20,788 (3.8) |
|  | n  % (95% CI) | n  % (95% CI) | n  % (95% CI) | n  % (95% CI) | n  % (95% CI) |
| ***Wider determinants of health*** |  |  |  |  |  |
| Unemployed and seeking work (n = 405,650) | 17,254 | 788 | 2,739 | 1,818 | 1,041 |
|  | 5.6 (5.5, 5.7) | 8.8 (8.2, 9.4) | 5.3 (5.1, 5.4) | 8.6 (8.3, 9.0) | 6.7 (6.3, 7.1) |
| Living in most deprived area (n = 549,552) | 52,062 | 2,091 | 12,737 | 6,573 | 3,895 |
|  | 12.3 (12.2, 12.4) | 17.9 (17.2, 18.6) | 19.2 (18.9, 19.5) | 25.1 (24.6, 25.6) | 18.7 (18.2, 19.3) |
| Complex social factors (n = 447,745) | 38,920 | 1,467 | 8,344 | 3,887 | 4,314 |
|  | 11.4 (11.3, 11.5) | 15.1 (14.4, 15.8) | 14.7 (14.5, 15.0) | 17.9 (17.4, 18.4) | 24.0 (23.4, 24.6) |
| English not as first language (n = 428,305) | 42,264 | 1,734 | 23,181 | 6,202 | 8,781 |
|  | 12.8 (12.7, 12.9) | 19.3 (18.5, 20.1) | 44.8 (44.4, 45.2) | 29.2 (28.6, 29.8) | 52.0 (51.3, 52.8) |
| ***Emotional and social health and support*** |  |  |  |  |  |
| No adequate support available during and after pregnancy (n = 386,808) | 19,442 | 407 | 1,414 | 780 | 670 |
|  | 6.6 (6.5, 6.7) | 5.0 (4.5, 5.5) | 2.9 (2.8, 3.1) | 4.0 (3.7, 4.3) | 4.5 (4.1, 4.8) |
| ***Reproductive health*** |  |  |  |  |  |
| Advanced maternal age (≥35 years) (n = 549,546) | 86,183 | 2,490 | 16,394 | 7,902 | 5,257 |
|  | 20.3 (20.2, 20.4) | 21.3 (20.6, 22.0) | 24.7 (24.3, 25.0) | 30.2 (29.6, 30.8) | 25.3 (24.7, 25.9) |
| Teenage pregnancy (<20 years) (n = 549,546) | 18,570 | 574 | 525 | 520 | 697 |
|  | 4.4 (4.3, 4.4) | 4.9 (4.5, 5.3) | 0.8 (0.7, 0.9) | 2.0 (1.8, 2.2) | 3.4 (3.1, 3.6) |
| Known previous obstetric complication (n = 290,396) | 50,652 | 1,628 | 12,295 | 5,000 | 2,689 |
|  | 23.0 (22.8, 23.2) | 25.5 (24.5, 26.6) | 33.6 (33.1, 34.1) | 29.7 (29.0, 30.4) | 25.9 (25.0, 26.7) |
| Previous pre-eclampsia, HELLP, eclampsia, gestational proteinuria (n = 290,396) | 2,254 | 74 | 363 | 295 | 71 |
|  | 1.0 (1.0, 1.1) | 1.2 (0.9, 1.5) | 1.0 (0.9, 1.1) | 1.8 (1.6, 2.0) | 0.7 (0.5, 0.9) |
| Previous gestational hypertension (n = 290,396) | 3,574 | 103 | 636 | 343 | 133 |
|  | 1.6 (1.6, 1.7) | 1.6 (1.3, 2.0) | 1.7 (1.6, 1.9) | 2.0 (1.8, 2.3) | 1.3 (1.1, 1.5) |
| Previous gestational diabetes mellitus (n = 290,396) | 3,679 | 140 | 2,149 | 530 | 289 |
|  | 1.7 (1.6, 1.7) | 2.2 (1.9, 2.6) | 5.9 (5.6, 6.1) | 3.1 (2.9, 3.4) | 2.8 (2.5, 3.1) |
| Previous caesarean section (n = 270,984) | 43,318 | 1,304 | 10,170 | 4,710 | 2,564 |
|  | 21.2 (21.0, 21.4) | 22.1 (21.0, 23.1) | 28.8 (28.3, 29.2) | 30.3 (29.6, 31.0) | 26.6 (25.8, 27.5) |
| Previous pregnancy loss (n = 266,477) | 80,345 | 2,678 | 11,013 | 6,534 | 3,419 |
|  | 39.9 (39.7, 40.2) | 44.5 (43.2, 45.8) | 31.9 (31.4, 32.4) | 41.6 (40.8, 42.3) | 37.5 (36.5, 38.5) |
| ***Health behaviours and weight*** |  |  |  |  |  |
| Not taken folic acid supplementation before pregnancy (n = 416,565) | 229,980 | 6,699 | 38,549 | 16,469 | 11,911 |
|  | 71.1 (71.0, 71.3) | 77.4 (76.5, 78.3) | 77.9 (77.5, 78.2) | 83.0 (82.5, 83.5) | 77.6 (77.0, 78.3) |
| Smoking around conception (n = 514,372) | 94,511 | 2,375 | 2,141 | 1,864 | 2,301 |
|  | 23.8 (23.7, 23.9) | 21.5 (20.7, 22.3) | 3.5 (3.3, 3.6) | 7.5 (7.2, 7.8) | 11.8 (11.3, 12.2) |
| Smokers who did not quit smoking during year before pregnancy (n = 120,830) | 94,511 | 2,375 | 2,141 | 1,864 | 2,301 |
|  | 85.8 (85.6, 86.0) | 83.8 (82.4, 85.1) | 79.2 (77.6, 80.7) | 79.0 (77.3, 80.6) | 82.7 (81.3, 84.1) |
| Underweight at booking (BMI <18.5 kg/m^2^) (n = 429,020) | 9,770 | 277 | 2,198 | 368 | 470 |
|  | 2.9 (2.9, 3.0) | 3.1 (2.7, 3.5) | 4.2 (4.1, 4.4) | 2.0 (1.8, 2.2) | 3.2 (3.0, 3.5) |
| Overweight at booking (BMI 25 to 29.9 kg/m^2^) (n = 429,020) | 91,004 | 2,572 | 16,595 | 6,306 | 4,247 |
|  | 27.2 (27,0, 27.3) | 28.7 (27.7, 29.6) | 32.0 (31.6, 32.4) | 33.9 (33.2, 34.6) | 29.1 (28.4, 29.9) |
| Obesity at booking (BMI ≥30 kg/m^2^) (n = 429,020) | 77,616 | 2,163 | 9,506 | 6,265 | 2,614 |
|  | 23.2 (23.0, 23.3) | 24.1 (23.2, 25.0) | 18.3 (18.0, 18.6) | 33.7 (33.0, 34.4) | 17.9 (17.3, 18.6) |
| ***Known pre-existing health conditions*** |  |  |  |  |  |
| At least one health condition (n = 549,552) | 116,850 | 2,963 | 11,518 | 5,656 | 3,453 |
|  | 27.5 (27.4, 27.7) | 25.3 (24.6, 26.1) | 17.3 (17.0, 17.6) | 21.6 (21.1, 22.1) | 16.6 (16.1, 17.1) |
| Mental health condition (n = 549,552) | 48,625 | 1,219 | 2,217 | 1,295 | 764 |
|  | 11.5 (11.4, 11.6) | 10.4 (9.9, 11.0) | 3.3 (3.2, 3.5) | 4.9 (4.7, 5.2) | 3.7 (3.4, 3.9) |
| Physical health condition (n = 549,552) | 89,785 | 2,298 | 10,260 | 4,999 | 3,001 |
|  | 21.2 (21.0, 21.3) | 19.6 (18.9, 20.4) | 15.4 (15.2, 15.7) | 19.1 (18.6, 19.6) | 14.4 (14.0, 14.9) |
| Diabetes (n = 549,552) | 3,575 | 118 | 1,268 | 387 | 194 |
|  | 0.8 (0.8, 0.9) | 1.0 (0.8, 1.2) | 1.9 (1.8, 2.0) | 1.5 (1.3, 1.6) | 0.9 (0.8, 1.1) |
| Hypertension (n = 549,552) | 4,575 | 132 | 679 | 548 | 174 |
|  | 1.1 (1.0, 1.1) | 1.1 (1.0, 1.3) | 1.0 (0.9, 1.1) | 2.1 (1.9, 2.3) | 0.8 (0.7, 1.0) |
| Cardiac disease (n = 549,552) | 4,044 | 79 | 224 | 142 | 118 |
|  | 1.0 (0.9, 1.0) | 0.7 (0.5, 0.8) | 0.3 (0.3, 0.4) | 0.5 (0.5, 0.6) | 0.6 (0.5, 0.7) |
| Thromboembolic condition (n = 549,552) | 3,036 | 60 | 207 | 121 | 119 |
|  | 0.7 (0.7, 0.7) | 0.5 (0.4, 0.7) | 0.3 (0.3, 0.4) | 0.5 (0.4, 0.5) | 0.6 (0.5, 0.7) |
| Renal disease (n = 549,552) | 3,975 | 83 | 275 | 91 | 152 |
|  | 0.9 (0.9, 1.0) | 0.7 (0.6, 0.9) | 0.4 (0.4, 0.5) | 0.3 (0.3, 0.4) | 0.7 (0.6, 0.9) |
| Hepatitis B (n = 549,552) | 335 | 20 | 202 | 221 | 87 |
|  | 0.1 (0.1, 0.1) | 0.2 (0.1, 0.3) | 0.3 (0.3, 0.3) | 0.8 (0.7, 1.0) | 0.4 (0.3, 0.5) |
| Cancer (n = 549,552) | 883 | 16 | 57 | 15 | 20 |
|  | 0.2 (0.2, 0.2) | 0.1 (0.1, 0.2) | 0.1 (0.1, 0.1) | 0.1 (0.0, 0.1) | 0.1 (0.1, 0.1) |
| ***Known family history*** |  |  |  |  |  |
| Inherited conditions (n = 549,552) | 10,390 | 237 | 814 | 266 | 194 |
|  | 2.4 (2.4, 2.5) | 2.0 (1.8, 2.3) | 1.2 (1.1, 1.3) | 1.0 (0.9, 1.1) | 0.9 (0.8, 1.1) |
| Diabetes (n = 549,552) | 77,218 | 2,993 | 25,921 | 6,447 | 4,892 |
|  | 18.2 (18.1, 18.3) | 25.6 (24.8, 26.4) | 39.0 (38.6, 39.4) | 24.6 (24.1, 25.2) | 23.5 (23.0, 24.1) |

BMI, body mass index; CI, confidence interval; HELLP, Elevated Liver enzymes and Low Platelet count

^1^ Definitions of indicators can be found in Supplementary Table 1.

**Table S5.** Unadjusted prevalence of preconception indicators by area-based level of deprivation, N = 652,880

|  | **Area-based level of deprivation (quintiles)** | | | | |
| --- | --- | --- | --- | --- | --- |
| **Preconception indicator**^1^ | **Most deprived**  **Quintile 1** | **Quintile 2** | **Quintile 3** | **Quintile 4** | **Least deprived Quintile 5** |
|  | 174,925 (26.8) | 147,740 (22.6) | 124,847 (19.1) | 110,983 (17.0) | 94,385 (14.5) |
|  | n  % (95% CI) | n  % (95% CI) | n  % (95% CI) | n  % (95% CI) | n  % (95% CI) |
| ***Wider determinants of health*** |  |  |  |  |  |
| Ethnic minority (n = 549,552) | 48,105 | 34,119 | 19,854 | 13,380 | 9,641 |
|  | 32.8 (32.5, 33.0) | 27.4 (27.1, 27.6) | 18.9 (18.7, 19.1) | 14.4 (14.2, 14.6) | 12.0 (11.8, 12.3) |
| Unemployed and seeking work (n = 472,181) | 12,909 | 6,623 | 3,729 | 2,230 | 1,358 |
|  | 10.7 (10.5, 10.8) | 5.9 (5.7, 6.0) | 4.0 (3.9, 4.1) | 2.8 (2.7, 2.9) | 2.0 (1.9, 2.2) |
| Complex social factors (n = 527,591) | 29,225 | 16,895 | 10,356 | 7,115 | 4,296 |
|  | 20.8 (20.6, 21.0) | 14.1 (13.9, 14.3) | 10.1 (10.0, 10.3) | 7.9 (7.7, 8.1) | 5.7 (5.5, 5.9) |
| English not as first language (n = 497,644) | 36,871 | 27,218 | 18,033 | 12,133 | 7,733 |
|  | 28.1 (27.8, 28.3) | 23.3 (23.1, 23.6) | 18.7 (18.4, 18.9) | 14.5 (14.3, 14.8) | 11.1 (10.9, 11.4) |
| ***Emotional and social health and support*** |  |  |  |  |  |
| No adequate support available during and after pregnancy (n = 449,884) | 6,301 | 5,157 | 4,841 | 5,038 | 5,253 |
|  | 5.1 (5.0, 5.2) | 4.8 (4.7, 5.0) | 5.6 (5.4, 5.7) | 6.8 (6.6, 7.0) | 8.8 (8.6, 9.0) |
| ***Reproductive health*** |  |  |  |  |  |
| Advanced maternal age (≥35 years) (n = 652,871) | 28,823 | 28,993 | 27,770 | 27,133 | 26,942 |
|  | 16.5 (16.3, 16.7) | 19.6 (19.4, 19.8) | 22.2 (22.0, 22.5) | 24.4 (24.2, 24.7) | 28.5 (28.3, 28.8) |
| Teenage pregnancy (<20 years) (n = 652,871) | 11,142 | 5,923 | 3,706 | 2,441 | 1,463 |
|  | 6.4 (6.3, 6.5) | 4.0 (3.9, 4.1) | 3.0 (2.9, 3.1) | 2.2 (2.1, 2.3) | 1.6 (1.5, 1.6) |
| Known previous obstetric complication (n = 329,228) | 24,415 | 18,040 | 14,714 | 12,470 | 11,055 |
|  | 26.4 (26.2, 26.7) | 24.2 (23.9, 24.5) | 23.8 (23.5, 24.2) | 22.9 (22.6, 23.3) | 23.9 (23.5, 24.3) |
| Previous pre-eclampsia, HELLP, eclampsia, gestational proteinuria (n = 329,228) | 1,193 | 759 | 595 | 497 | 425 |
|  | 1.3 (1.2, 1.4) | 1.0 (0.9, 1.1) | 1.0 (0.9, 1.0) | 0.9 (0.8, 1.0) | 0.9 (0.8, 1.0) |
| Previous gestational hypertension (n = 329,228) | 1,667 | 1,227 | 921 | 734 | 659 |
|  | 1.8 (1.7, 1.9) | 1.6 (1.6, 1.7) | 1.5 (1.4, 1.6) | 1.3 (1.3, 1.4) | 1.4 (1.3, 1.5) |
| Previous gestational diabetes mellitus (n = 329,228) | 2,630 | 1,709 | 1,302 | 1,020 | 845 |
|  | 2.8 (2.7, 3.0) | 2.3 (2.2, 2.4) | 2.1 (2.0, 2.2) | 1.9 (1.8, 2.0) | 1.8 (1.7, 2.0) |
| Previous caesarean section (n = 306,430) | 20,131 | 15,939 | 12,919 | 11,290 | 9,711 |
|  | 23.3 (23.0, 23.6) | 23.2 (22.9, 23.5) | 22.4 (22.1, 22.8) | 22.2 (21.9, 22.6) | 22.6 (22.2, 23.0) |
| Previous pregnancy loss (n = 301,168) | 31,891 | 26,591 | 22,338 | 19,610 | 16,828 |
|  | 37.8 (37.5, 38.2) | 39.2 (38.8, 39.6) | 39.5 (39.1, 39.9) | 39.3 (38.8, 39.7) | 39.5 (39.1, 40.0) |
| ***Health behaviours and weight*** |  |  |  |  |  |
| Not taken folic acid supplementation before pregnancy (n = 488,987) | 106,823 | 86,979 | 67,399 | 53,766 | 40,681 |
|  | 83.0 (82.8, 83.2) | 75.5 (75.2, 75.7) | 70.3 (70.0, 70.6) | 65.5 (65.2, 65.9) | 60.6 (60.2, 60.9) |
| Smoking around conception (n = 604,514) | 44,429 | 28,663 | 20,156 | 14,833 | 9,521 |
|  | 27.8 (27.6, 28.0) | 20.8 (20.6, 21.0) | 17.3 (17.1, 17.5) | 14.4 (14.2, 14.7) | 10.9 (10.7, 11.1) |
| Smokers who did not quit smoking during year before pregnancy (n = 138,422) | 44,429 | 28,663 | 20,156 | 14,833 | 9,521 |
|  | 90.5 (90.2, 90.7) | 85.4 (85.0, 85.7) | 82.1 (81.6, 82.5) | 79.6 (79.0, 80.1) | 76.0 (75.2, 76.7) |
| Underweight at booking (BMI <18.5 kg/m^2^) (n = 496,331) | 4,632 | 3,544 | 2,679 | 2,432 | 2,059 |
|  | 3.6 (3.5, 3.8) | 3.1 (3.0, 3.2) | 2.8 (2.7, 2.9) | 2.8 (2.7, 2.9) | 2.8 (2.7, 2.9) |
| Overweight at booking (BMI 25 to 29.9 kg/m^2^) (n = 496,331) | 36,043 | 31,997 | 27,077 | 23,681 | 19,976 |
|  | 28.4 (28.1, 28.6) | 28.3 (28.1, 28.6) | 28.1 (27.8, 28.4) | 27.6 (27.3, 27.9) | 26.9 (26.6, 27.2) |
| Obesity at booking (BMI ≥30 kg/m^2^) (n = 496,331) | 35,383 | 26,690 | 20,241 | 16,278 | 12,036 |
|  | 27.9 (27.6, 28.1) | 23.6 (23.4, 23.9) | 21.0 (20.8, 21.3) | 19.0 (18.7, 19.2) | 16.2 (16.0, 16.5) |
| ***Known pre-existing health conditions*** |  |  |  |  |  |
| At least one health condition (n = 652,880) | 43,164 | 35,796 | 30,910 | 27,215 | 21,754 |
|  | 24.7 (24.5, 24.9) | 24.2 (24.0, 24.4) | 24.8 (24.5, 25.0) | 24.5 (24.3, 24.8) | 23.0 (22.8, 23.3) |
| Mental health condition (n = 652,880) | 19,107 | 14,174 | 11,466 | 9,458 | 6,768 |
|  | 10.9 (10.8, 11.1) | 9.6 (9.4, 9.7) | 9.2 (9.0, 9.3) | 8.5 (8.4, 8.7) | 7.2 (7.0, 7.3) |
| Physical health condition (n = 652,880) | 32,408 | 27,826 | 24,512 | 21,993 | 17,966 |
|  | 18.5 (18.3, 18.7) | 18.8 (18.6, 19.0) | 19.6 (19.4, 19.9) | 19.8 (19.6, 20.1) | 19.0 (18.8, 19.3) |
| Diabetes (n = 652,880) | 2,365 | 1,418 | 1,044 | 835 | 681 |
|  | 1.4 (1.3, 1.4) | 1.0 (0.9, 1.0) | 0.8 (0.8, 0.9) | 0.8 (0.7, 0.8) | 0.7 (0.7, 0.8) |
| Hypertension (n = 652,880) | 2,101 | 1,557 | 1,264 | 1,000 | 774 |
|  | 1.2 (1.2, 1.3) | 1.1 (1.0, 1.1) | 1.0 (1.0, 1.1) | 0.9 (0.8, 1.0) | 0.8 (0.8, 0.9) |
| Cardiac disease (n = 652,880) | 1,391 | 1,176 | 986 | 877 | 754 |
|  | 0.8 (0.8, 0.8) | 0.8 (0.8, 0.8) | 0.8 (0.7, 0.8) | 0.8 (0.7, 0.8) | 0.8 (0.7, 0.9) |
| Thromboembolic condition (n = 652,880) | 1,052 | 878 | 729 | 714 | 512 |
|  | 0.6 (0.6, 0.6) | 0.6 (0.6, 0.6) | 0.6 (0.5, 0.6) | 0.6 (0.6, 0.7) | 0.5 (0.5, 0.6) |
| Renal disease (n = 652,880) | 1,191 | 1,098 | 1,114 | 939 | 784 |
|  | 0.7 (0.6, 0.7) | 0.7 (0.7, 0.8) | 0.9 (0.8, 0.9) | 0.8 (0.8, 0.9) | 0.8 (0.8, 0.9) |
| Hepatitis B (n = 652,880) | 400 | 265 | 162 | 92 | 79 |
|  | 0.2 (0.2, 0.3) | 0.2 (0.2, 0.2) | 0.1 (0.1, 0.2) | 0.1 (0.1, 0.1) | 0.1 (0.1, 0.1) |
| Cancer (n = 652,880) | 193 | 228 | 262 | 184 | 218 |
|  | 0.1 (0.1, 0.1) | 0.2 (0.1, 0.2) | 0.2 (0.2, 0.2) | 0.2 (0.1, 0.2) | 0.2 (0.2, 0.3) |
| ***Known family history*** |  |  |  |  |  |
| Inherited conditions (n = 652,880) | 3,281 | 3,012 | 2,667 | 2,379 | 1,984 |
|  | 1.9 (1.8, 1.9) | 2.0 (2.0, 2.1) | 2.1 (2.1, 2.2) | 2.1 (2.1, 2.2) | 2.1 (2.0, 2.2) |
| Diabetes (n = 652,880) | 41,705 | 32,558 | 24,406 | 19,928 | 15,801 |
|  | 23.8 (23.6, 24.0) | 22.0 (21.8, 22.2) | 19.5 (19.3, 19.8) | 18.0 (17.7, 18.2) | 16.7 (16.5, 17.0) |

BMI, body mass index; CI, confidence interval; HELLP, Elevated Liver enzymes and Low Platelet count

^1^ Definitions of indicators can be found in Supplementary Table 1.

**Table S6.** Unadjusted prevalence of preconception indicators by previous pregnancy, N = 524,572

|  | **Previous pregnancy** | | |
| --- | --- | --- | --- |
| **Preconception indicator**^1^ | **Previous livebirth (with or without pregnancy loss)** | **Previous pregnancy loss (no livebirth)** | **No previous pregnancy** |
|  | 288,073 (54.9) | 37,550 (7.2) | 198,949 (37,9) |
|  | n  % (95% CI) | n  % (95% CI) | n  % (95% CI) |
| ***Wider determinants of health*** |  |  |  |
| Ethnic minority (n = 450,008) | 62,716 | 6,796 | 38,120 |
|  | 24.6 (24.4, 24.7) | 21.1 (20.6, 21.5) | 23.5 (23.3, 23.7) |
| Unemployed and seeking work (n = 425,401) | 14,504 | 1,874 | 8,806 |
|  | 6.2 (6.1, 6.3) | 6.0 (5.7, 6.2) | 5.5 (5.4, 5.6) |
| Living in most deprived area (n = 524,572) | 44,279 | 4,249 | 22,989 |
|  | 15.4 (15.2, 15.5) | 11.3 (11.0, 11.6) | 11.6 (11.4, 11.7) |
| Complex social factors (n = 447,830) | 28,952 | 4,268 | 24,676 |
|  | 11.9 (11.8, 12.0) | 13.1 (12.8, 13.5) | 14.3 (14.2, 14.5) |
| English not as first language (n = 436,012) | 53,000 | 5,985 | 34,219 |
|  | 21.6 (21.5, 21.8) | 18.8 (18.4, 19.2) | 21.5 (21.3, 21.7) |
| ***Emotional and social health and support*** |  |  |  |
| No adequate support available during and after pregnancy (n = 401,862) | 11,842 | 1,920 | 12,087 |
|  | 5.3 (5.2, 5.4) | 6.6 (6.4, 6.9) | 8.0 (7.8, 8.1) |
| ***Reproductive health*** |  |  |  |
| Advanced maternal age (≥35 years) (n = 524,564) | 79,607 | 7,596 | 27,792 |
|  | 27.6 (27.5, 27.8) | 20.2 (19.8, 20.6) | 14.0 (13.8, 14.1) |
| Teenage pregnancy (<20 years) (n = 524,564) | 2,889 | 1,739 | 13,905 |
|  | 1.0 (1.0, 1.0) | 4.6 (4.4, 4.8) | 7.0 (6.9, 7.1) |
| ***Health behaviours and weight*** |  |  |  |
| Not taken folic acid supplementation before pregnancy (n = 422,534) | 178,559 | 19,422 | 111,462 |
|  | 77.1 (76.9, 77.3) | 64.8 (64.3, 65.4) | 69.3 (69.0, 69.5) |
| Smoking around conception (n = 504,934) | 56,397 | 7,487 | 33,577 |
|  | 20.2 (20.1, 20.4) | 20.7 (20.2, 21.1) | 17.7 (17.5, 17.8) |
| Smokers who did not quit smoking during year before pregnancy (n = 115,925) | 56,397 | 7,487 | 33,577 |
|  | 87.1 (86.9, 87.4) | 76.8 (75.9, 77.6) | 81.0 (80.6, 81.4) |
| Underweight at booking (BMI <18.5 kg/m^2^) (n = 421,712) | 6,408 | 970 | 5,765 |
|  | 2.7 (2.7, 2.8) | 3.1 (2.9, 3.3) | 3.7 (3.6, 3.8) |
| Overweight at booking (BMI 25 to 29.9 kg/m^2^) (n = 421,712) | 68,457 | 8,876 | 40,789 |
|  | 29.1 (28.9, 29.3) | 28.1 (27.6, 28.6) | 26.3 (26.1, 26.6) |
| Obesity at booking (BMI ≥30 kg/m^2^) (n = 421,712) | 59,521 | 6,701 | 28,524 |
|  | 25.3 (25.1, 25.5) | 21.2 (20.7, 21.6) | 18.4 (18.2, 18.6) |
| ***Known pre-existing health conditions*** |  |  |  |
| At least one health condition (n = 524,572) | 82,327 | 11,658 | 48,853 |
|  | 28.6 (28.4, 28.7) | 31.0 (30.6, 31.5) | 24.6 (24.4, 24.7) |
| Mental health condition (n = 524,572) | 32,777 | 4,674 | 17,766 |
|  | 11.4 (11.3, 11.5) | 12.4 (12.1, 12.8) | 8.9 (8.8, 9.1) |
| Physical health condition (n = 524,572) | 64,443 | 9,189 | 38,576 |
|  | 22.4 (22.2, 22.5) | 24.5 (24.0, 24.9) | 19.4 (19.2, 19.6) |
| Diabetes (n = 524,572) | 4,059 | 425 | 1,241 |
|  | 1.4 (1.4, 1.5) | 1.1 (1.0, 1.2) | 0.6 (0.6, 0.7) |
| Hypertension (n = 524,572) | 4,786 | 296 | 1,000 |
|  | 1.7 (1.6, 1.7) | 0.8 (0.7, 0.9) | 0.5 (0.5, 0.5) |
| Cardiac disease (n = 524,572) | 2,570 | 386 | 1,698 |
|  | 0.9 (0.9, 0.9) | 1.0 (0.9, 1.1) | 0.9 (0.8, 0.9) |
| Thromboembolic condition (n = 524,572) | 2,360 | 183 | 929 |
|  | 0.8 (0.8, 0.9) | 0.5 (0.4, 0.6) | 0.5 (0.4, 0.5) |
| Renal disease (n = 524,572) | 2,630 | 374 | 1,651 |
|  | 0.9 (0.9, 0.9) | 1.0 (0.9, 1.1) | 0.8 (0.8, 0.9) |
| Hepatitis B (n = 524,572) | 616 | 62 | 258 |
|  | 0.2 (0.2, 0.2) | 0.2 (0.1, 0.2) | 0.1 (0.1, 0.1) |
| Cancer (n = 524,572) | 561 | 84 | 316 |
|  | 0.2 (0.2, 0.2) | 0.2 (0.2, 0.3) | 0.2 (0.1, 0.2) |
| ***Known family history*** |  |  |  |
| Inherited conditions (n = 524,572) | 6,778 | 924 | 4,589 |
|  | 2.4 (2.3, 2.4) | 2.5 (2.3, 2.6) | 2.3 (2.2, 2.4) |
| Diabetes (n = 524,572) | 69,213 | 9,505 | 45,147 |
|  | 24.0 (23.9, 24.2) | 25.3 (24.9, 25.8) | 22.7 (22.5, 22.9) |

BMI, body mass index; CI, confidence interval; HELLP, Elevated Liver enzymes and Low Platelet count

^1^ Definitions of indicators can be found in Supplementary Table 1.

**Table S7.** Ranking of preconception indicators by members of the UK Preconception Partnership, N = 27

| **Preconception indicator** | **Mean score** | **Ranking** |
| --- | --- | --- |
| Not taken folic acid supplementation before pregnancy | 6.7 | 1 |
| Obesity | 6.3 | 2 |
| Complex social factors | 6.1 | 3 |
| Living in most deprived area (bottom 10%) | 5.5 | 4 |
| Smoking around the time of conception | 5.3 | 5 |
| Overweight at booking | 3.6 | 6 |
| Pre-existing mental health condition | 3.4 | 7 |
| Pre-existing physical health condition | 3.1 | 8 |
| Previous pregnancy loss | 3.0 | 9 |
| Previous obstetric complication | 2.9 | 10 |
| Pre-existing mental and/or physical health condition | 2.3 | 11 |
| Not quit smoking during year before pregnancy | 2.0 | 12 |
| Advanced maternal age at booking | 1.9 | 13 |
| Unemployed and seeking work | 1.9 | 14 |
| Previous caesarean section | 0.3 | 15 |

**Table S8.** Unadjusted and adjusted prevalence of 10 prioritised preconception indicators by maternal age

|  |  | **Maternal age (years)** | | | | | | |
| --- | --- | --- | --- | --- | --- | --- | --- | --- |
| **Preconception indicator** |  | **<20** | **20-24** | **25-29** | **30-34** | **35-39** | **40+** |  |
|  |  | n  % (95% CI) | n  % (95% CI) | n  % (95% CI) | n  % (95% CI) | n  % (95% CI) | n  % (95% CI) |  |
| ***Wider determinants of health*** |  |  |  |  |  |  |  |  |
| Living in most deprived area | Unadjusted  (n = 652,871) | 6,746 | 20,756 | 28,167 | 22,739 | 11,313 | 2,807 |  |
|  |  | 27.3 (26.8, 27.9) | 21.4 (21.1, 21.6) | 15.3 (15.1, 15.5) | 11.0 (10.8, 11.1) | 10.0 (9.8, 10.1) | 10.7 (10.3, 11.1) |  |
|  | Unadjusted  (n = 450,002) | 4,000 | 13,153 | 18,382 | 15,391 | 7,800 | 1,966 |  |
|  |  | 25.0 (24.3, 25.7) | 20.0 (19.7, 20.3) | 14.6 (14.4, 14.8) | 10.8 (10.6, 10.9) | 9.7 (9.5, 9.9) | 10.5 (10.0, 10.9) |  |
|  | Adjusted^1^  (n = 450,002) | 29.1 (27.6, 30.7) | 21.4 (21.1, 21.7) | 15.0 (14.8, 15.2) | 10.3 (10.2, 10.5) | 8.8 (8.6, 9.0) | 8.9 (8.5, 9.3) |  |
| Complex social factors | Unadjusted  (n = 527,583) | 16,140 | 11,768 | 16,007 | 14,432 | 7,484 | 2,056 |  |
|  |  | 80.5 (80.0, 81.0) | 15.3 (15.0, 15.5) | 10.8 (10.7, 11.0) | 8.6 (8.4, 8.7) | 8.0 (7.9, 8.2) | 9.5 (9.2, 9.9) |  |
|  | Unadjusted  (n = 383,833) | 11,128 | 8,420 | 11,599 | 10,758 | 5,584 | 1,564 |  |
|  |  | 79.3 (78.7, 80.0) | 15.2 (14.9, 15.5) | 10.9 (10.7, 11.1) | 8.8 (8.6, 9.0) | 8.1 (7.9, 8.3) | 9.7 (9.3, 10.2) |  |
|  | Adjusted^2^  (n = 383,833) | 78.9 (77.5, 80.3) | 15.1 (14.8, 15.5) | 10.7 (10.6, 10.9) | 9.0 (8.8, 9.1) | 8.2 (8.0, 8.4) | 9.3 (8.9, 9.8) |  |
| ***Reproductive health*** |  |  |  |  |  |  |  |  |
| Known previous obstetric complication | Unadjusted  (n = 329,224) | 581 | 6,970 | 19,225 | 28,276 | 20,454 | 5,188 |  |
|  |  | 12.2 (11.3, 13.1) | 17.9 (17.5, 18.3) | 22.4 (22.1, 22.6) | 25.4 (25.1, 25.6) | 28.9 (28.5, 29.2) | 30.2 (29.6, 30.9) |  |
|  | Unadjusted  (n = 290,394) | 524 | 6,227 | 17,203 | 25,318 | 18,335 | 4,657 |  |
|  |  | 12.5 (11.4, 13.5) | 18.0 (17.6, 18.4) | 22.6 (22.3, 23.0) | 25.8 (25.6, 26.1) | 29.4 (29.1, 29.8) | 30.9 (30.1, 31.6) |  |
|  | Adjusted^3^  (n = 290,394) | 11.8 (10.4, 13.2) | 18.1 (17.6, 18.6) | 22.7 (22.4, 23.0) | 26.0 (25.7, 26.2) | 29.4 (29.0, 29.8) | 30.8 (30.0, 31.5) |  |
| Previous pregnancy loss | Unadjusted  (n = 301,164) | 2,352 | 14,770 | 29,782 | 37,010 | 25,685 | 7,656 |  |
|  |  | 52.8 (51.3, 54.2) | 41.3 (40.8, 41.8) | 37.9 (37.6, 38.3) | 36.4 (36.1, 36.7) | 39.6 (39.2, 40.0) | 48.4 (47.6, 49.2) |  |
|  | Unadjusted  (n = 266,475) | 2,089 | 13,170 | 26,520 | 32,680 | 22,739 | 6,789 |  |
|  |  | 53.3 (48.7, 57.9) | 41.4 (40.8, 41.9) | 38.1 (37.7, 38.4) | 36.4 (36.1, 36.7) | 39.7 (39.3, 40.1) | 48.7 (47.8, 49.5) |  |
|  | Adjusted^3^  (n = 266,475) | 55.2 (53.0, 57.4) | 40.8 (40.2, 41.4) | 37.4 (37.0, 37.7) | 36.1 (35.8, 36.4) | 39.4 (39.0, 39.9) | 48.4 (47.5, 49.2) |  |
| ***Health behaviours and weight*** |  |  |  |  |  |  |  |  |
| Not taken folic acid supplementation before pregnancy | Unadjusted  (n = 488,980) | 16,720 | 62,678 | 104,715 | 104,278 | 54,748 | 12,504 |  |
|  |  | 91.6 (91.1, 92.0) | 85.8 (85.6, 86.1) | 75.4 (75.2, 75.6) | 67.3 (67.0, 67.5) | 64.9 (64.6, 65.2) | 64.5 (63.8, 65.2) |  |
|  | Unadjusted  (n = 360993) | 11,932 | 46,054 | 77,543 | 77,715 | 41,621 | 9,586 |  |
|  |  | 91.9 (91.4, 92.4) | 86.4 (86.1, 86.7) | 76.0 (75.8, 76.3) | 67.9 (67.7, 68.2) | 65.5 (65.1, 65.9) | 65.0 (64.3, 65.8) |  |
|  | Adjusted^2^  (n = 360,993) | 91.1 (90.0, 92.2) | 86.4 (86.0, 86.7) | 76.3 (76.0, 76.5) | 67.9 (67.7, 68.2) | 64.6 (64.2, 65.0) | 62.7 (61.8, 63.6) |  |
| Smoking around conception | Unadjusted  (n = 604,506) | 10,145 | 30,034 | 36,252 | 26,225 | 12,270 | 2,675 |  |
|  |  | 44.9 (44.2, 45.5) | 33.5 (33.2, 33.8) | 21.3 (21.1, 21.5) | 13.6 (13.5, 13.8) | 11.6 (11.4, 11.8) | 11.0 (10.6, 11.4) |  |
|  | Unadjusted  (n = 435,059) | 7,093 | 21,948 | 27,127 | 19,300 | 8,918 | 1,964 |  |
|  |  | 46.0 (45.2, 46.7) | 34.5 (34.2, 34.9) | 22.3 (22.1, 22.5) | 13.9 (13.8, 14.1) | 11.5 (11.2, 11.7) | 10.8 (10.4, 11.3) |  |
|  | Adjusted^2^  (n = 435,059) | 40.8 (39.4, 42.3) | 31.8 (31.4, 32.2) | 21.7 (21.5, 22.0) | 14.9 (14.7, 15.1) | 12.8 (12.5, 13.0) | 12.3 (11.8, 12.9) |  |
| Overweight at booking (BMI 25 to 29.9 kg/m^2^) | Unadjusted  (n = 496,327) | 3,787 | 18,354 | 39,084 | 45,510 | 25,887 | 6,150 |  |
|  |  | 22.7 (22.1, 23.3) | 25.8 (25.5, 26.1) | 27.8 (27.6, 28.0) | 28.3 (28.1, 28.5) | 29.5 (29.2, 29.8) | 31.4 (30.7, 32.0) |  |
|  | Unadjusted  (n = 368,258) | 2,671 | 13,349 | 29,066 | 34,095 | 19,865 | 4,683 |  |
|  |  | 22.7 (21.8, 23.3) | 25.7 (25.2, 25.9) | 28.1 (27.8, 28.3) | 28.6 (28.2, 28.7) | 29.8 (29.4, 30.1) | 31.2 (30.4, 31.8) |  |
|  | Adjusted^2^  (n = 368,258) | 22.3 (20.6, 24.0) | 25.9 (25.5, 26.4) | 28.1 (27.8, 28.4) | 28.3 (28.1, 28.6) | 29.5 (29.1, 29.9) | 30.8 (29.9, 31.6) |  |
| Obesity at booking (BMI ≥30 kg/m^2^) | Unadjusted  (n = 496,327) | 2,807 | 17,687 | 33,207 | 33,023 | 18,969 | 4,933 |  |
|  |  | 16.8 (16.3, 17.4) | 24.9 (24.5, 25.2) | 23.6 (23.4, 23.8) | 20.6 (20.4, 20.7) | 21.6 (21.4, 21.9) | 25.2 (24.6, 25.8) |  |
|  | Unadjusted  (n = 368,258) | 1,984 | 13,211 | 25,284 | 25,473 | 14,866 | 3,925 |  |
|  |  | 16.8 (16.2, 17.5) | 25.4 (25.0, 25.8) | 24.4 (24.1, 24.7) | 21.4 (21.1, 21.6) | 22.3 (22.0, 22.6) | 26.2 (25.5, 26.9) |  |
|  | Adjusted^2^  (n = 368,258) | 19.1 (17.6, 20.7) | 25.1 (24.6, 25.5) | 24.3 (24.1, 24.6) | 21.7 (21.5, 22.0) | 22.6 (22.3, 23.0) | 25.3 (24.5, 26.0) |  |
| ***Known pre-existing health conditions*** |  |  |  |  |  |  |  |  |
| Mental health condition | Unadjusted  (n = 652,871) | 3,618 | 11,684 | 17,286 | 16,713 | 9,472 | 2,198 |  |
|  |  | 14.7 (14.2, 15.1) | 12.0 (11.8, 12.2) | 9.4 (9.3, 9.5) | 8.1 (7.9, 8.2) | 8.3 (8.2, 8.5) | 8.4 (8.1, 8.7) |  |
|  | Unadjusted  (n = 450,002) | 2,872 | 9,399 | 13,856 | 13,520 | 7,643 | 1,767 |  |
|  |  | 18.0 (17.4, 18.5) | 14.3 (14.0, 14.6) | 11.0 (10.8, 11.2) | 9.4 (9.3, 9.6) | 9.5 (9.3, 9.7) | 9.4 (9.0, 9.8) |  |
|  | Adjusted^2^  (n = 450,002) | 16.7 (15.6, 17.8) | 13.5 (13.2, 13.7) | 10.9 (10.7, 11.0) | 9.8 (9.6, 9.9) | 9.9 (9.7, 10.1) | 10.0 (9.5, 10.5) |  |
| Physical health condition | Unadjusted  (n = 652,871) | 2,749 | 14,648 | 32,951 | 41,839 | 25,877 | 6,641 |  |
|  |  | 11.1 (10.8, 11.5) | 15.1 (14.8, 15.3) | 17.9 (17.7, 18.1) | 20.2 (20.0, 20.4) | 22.8 (22.6, 23.1) | 25.3 (24.8, 25.9) |  |
|  | Unadjusted  (n = 450,002) | 2,199 | 11,762 | 26,175 | 33,183 | 20,779 | 5,363 |  |
|  |  | 13.7 (13.2, 14.3) | 17.9 (17.6, 18.2) | 20.8 (20.6, 21.0) | 23.2 (23.0, 23.4) | 25.8 (25.5, 26.1) | 28.6 (27.9, 29.2) |  |
|  | Adjusted^2^  (n = 450,002) | 13.1 (12.1, 14.1) | 17.4 (17.0, 17.7) | 20.7 (20.5, 20.9) | 23.4 (23.2, 23.6) | 26.1 (25.8, 26.4) | 28.6 (27.9, 29.4) |  |

^1^ Adjusted for ethnicity and previous pregnancy

^2^ Adjusted for ethnicity, area-based level of deprivation and previous pregnancy

^3^ Adjusted for ethnicity and area-based level of deprivation

**Table S9.** Unadjusted and adjusted prevalence of 10 prioritised preconception indicators by ethnicity

|  |  | **Ethnicity** | | | | |
| --- | --- | --- | --- | --- | --- | --- |
| **Preconception indicator** |  | **White** | **Mixed** | **Asian** | **Black** | **Other** |
|  |  | n  % (95% CI) | n  % (95% CI) | n  % (95% CI) | n  % (95% CI) | n  % (95% CI) |
| ***Wider determinants of health*** |  |  |  |  |  |  |
| Living in most deprived area | Unadjusted  (n = 549,552) | 52,062 | 2,091 | 12,737 | 6,573 | 3,895 |
|  |  | 12.3 (12.2, 12.4) | 17.9 (17.2, 18.6) | 19.2 (18.9, 19.5) | 25.1 (24.6, 25.6) | 18.7 (18.2, 19.3) |
|  | Unadjusted  (n = 450,002) | 39,928 | 1,681 | 10,497 | 5,592 | 2,994 |
|  |  | 11.7 (11.6, 11.8) | 17.2 (16.4, 17.9) | 18.2 (17.8, 18.5) | 24.3 (23.7, 24.9) | 17.6 (17.0, 18.2) |
|  | Adjusted  (n = 450,002)^1^ | 11.4 (11.3, 11.5) | 17.0 (16.2, 17.7) | 19.1 (18.8, 19.5) | 24.3 (23.7, 24.9) | 18.3 (17.7, 18.9) |
| Complex social factors | Unadjusted  (n = 447,745) | 38,920 | 1,467 | 8,344 | 3,887 | 4,314 |
|  |  | 11.4 (11.3, 11.5) | 15.1 (14.4, 15.8) | 14.7 (14.5, 15.0) | 17.9 (17.4, 18.4) | 24.0 (23.4, 24.6) |
|  | Unadjusted  (n = 383,833) | 33,134 | 1,242 | 7,608 | 3,456 | 3,613 |
|  |  | 11.5 (11.3, 11.6) | 14.7 (13.9, 15.5) | 14.8 (14.5, 15.1) | 17.5 (17.0, 18.1) | 23.5 (22.8, 24.2) |
|  | Adjusted  (n = 383,833)^2^ | 11.3 (11.2, 11.4) | 13.0 (12.4, 13.7) | 16.2 (15.8, 16.5) | 17.0 (16.4, 17.6) | 22.9 (22.3, 23.6) |
| ***Reproductive health*** |  |  |  |  |  |  |
| Known previous obstetric complication | Unadjusted  (n = 290,396) | 50,652 | 1,628 | 12,295 | 5,000 | 2,689 |
|  |  | 23.0 (22.8, 23.2) | 25.5 (24.5, 26.6) | 33.6 (33.1, 34.1) | 29.7 (29.0, 30.4) | 25.9 (25.0, 26.7) |
|  | Unadjusted  (n = 290,394) | 50,652 | 1,628 | 12,295 | 5,000 | 2,689 |
|  |  | 23.0 (22.8, 23.2) | 25.5 (24.5, 26.6) | 33.6 (33.1, 34.1) | 29.7 (29.0, 30.4) | 25.9 (25.0, 26.7) |
|  | Adjusted^3^  (n = 290,394) | 23.6 (23.4, 23.7) | 25.5 (24.5, 26.6) | 32.2 (31.7, 32.8) | 28.0 (27.2, 28.9) | 25.2 (24.3, 26.0) |
| Previous pregnancy loss | Unadjusted  (n = 266,477) | 80,345 | 2,678 | 11,013 | 6,534 | 3,419 |
|  |  | 39.9 (39.7, 40.2) | 44.5 (43.2, 45.8) | 31.9 (31.4, 32.4) | 41.6 (40.8, 42.3) | 37.5 (36.5, 38.5) |
|  | Unadjusted  (n = 265,807) | 80,343 | 2,678 | 11,013 | 6,534 | 3,419 |
|  |  | 39.9 (39.7, 40.1) | 45.0 (43.7, 46.3) | 32.1 (31.6, 32.7) | 42.2 (41.2, 43.2) | 38.2 (37.1, 39.2) |
|  | Adjusted^3^  (n = 265,807) | 39.4 (39.2, 39.6) | 43.5 (42.2, 44.8) | 32.3 (31.8, 32.9) | 41.0 (40.0, 42.0) | 36.4 (35.3, 37.4) |
| ***Health behaviours and weight*** |  |  |  |  |  |  |
| Not taken folic acid supplementation before pregnancy | Unadjusted  (n = 416,565) | 229,980 | 6,699 | 38,549 | 16,469 | 11,911 |
|  |  | 71.1 (71.0, 71.3) | 77.4 (76.5, 78.3) | 77.9 (77.5, 78.2) | 83.0 (82.5, 83.5) | 77.6 (77.0, 78.3) |
|  | Unadjusted  (n = 360,993) | 197,112 | 5,910 | 35,792 | 15,162 | 10,475 |
|  |  | 71.4 (71.2, 71.6) | 77.6 (76.6, 78.5) | 78.2 (77.8, 78.6) | 83.5 (83.0, 84.1) | 78.1 (77.4, 78.8) |
|  | Adjusted^2^  (n = 360,993) | 71.7 (71.6, 71.9) | 76.4 (75.5, 77.4) | 77.3 (76.9, 77.7) | 80.9 (80.1, 81.7) | 77.9 (77.2, 78.7) |
| Smoking around conception | Unadjusted  (n = 514,372) | 94,511 | 2,375 | 2,141 | 1,864 | 2,301 |
|  |  | 23.8 (23.7, 23.9) | 21.5 (20.7, 22.3) | 3.5 (3.3, 3.6) | 7.5 (7.2, 7.8) | 11.8 (11.3, 12.2) |
|  | Unadjusted  (n = 435,059) | 78,834 | 2,020 | 1,947 | 1,652 | 1,897 |
|  |  | 23.9 (23.7, 24.0) | 21.3 (20.5, 22.1) | 3.5 (3.3, 3.6) | 7.4 (7.0, 7.7) | 11.5 (11.0, 12.0) |
|  | Adjusted^2^  (n = 435,059) | 24.3 (24.2, 24.4) | 19.7 (19.0, 20.5) | 3.8 (3.6, 4.0) | 7.5 (7.1, 8.0) | 11.2 (10.7, 11.7) |
| Overweight at booking (BMI 25 to 29.9 kg/m^2^) | Unadjusted  (n 429,020) | 91,004 | 2,572 | 16,595 | 6,306 | 4,247 |
|  |  | 27.2 (27,0, 27.3) | 28.7 (27.7, 29.6) | 32.0 (31.6, 32.4) | 33.9 (33.2, 34.6) | 29.1 (28.4, 29.9) |
|  | Unadjusted  (n = 368,258) | 76,744 | 2,232 | 15,318 | 5,776 | 3,659 |
|  |  | 27.1 (26.8, 27.1) | 28.9 (27.9, 29.9) | 32.1 (31.7, 32.4) | 34.0 (33.2, 34.6) | 29.3 (28.6, 30.0) |
|  | Adjusted^2^  (n = 368,258) | 27.0 (26.9, 27.2) | 28.7 (27.7, 29.7) | 30.6 (30.2, 31.1) | 33.8 (32.9, 34.8) | 28.6 (27.8, 29.5) |
| Obesity at booking (BMI ≥30 kg/m^2^) | Unadjusted  (n = 429,020) | 77,616 | 2,163 | 9,506 | 6,265 | 2,614 |
|  |  | 23.2 (23.0, 23.3) | 24.1 (23.2, 25.0) | 18.3 (18.0, 18.6) | 33.7 (33.0, 34.4) | 17.9 (17.3, 18.6) |
|  | Unadjusted  (n = 368,258) | 66,033 | 1,876 | 8,800 | 5,777 | 2,257 |
|  |  | 23.3 (23.1, 23.5) | 24.3 (23.3, 25.2) | 18.4 (18.1, 18.8) | 34.0 (33.3, 34.7) | 18.1 (17.4, 18.8) |
|  | Adjusted^2^  (n = 368,258) | 23.8 (23.6, 23.9) | 23.5 (22.5, 24.4) | 16.8 (16.4, 17.1) | 29.8 (29.0, 30.7) | 17.3 (16.6, 18.0) |
| ***Known pre-existing health conditions*** |  |  |  |  |  |  |
| Mental health condition | Unadjusted  (n = 549,552) | 48,625 | 1,219 | 2,217 | 1,295 | 764 |
|  |  | 11.5 (11.4, 11.6) | 10.4 (9.9, 11.0) | 3.3 (3.2, 3.5) | 4.9 (4.7, 5.2) | 3.7 (3.4, 3.9) |
|  | Unadjusted  (n = 450,002) | 43,868 | 1,106 | 2,129 | 1,251 | 703 |
|  |  | 12.8 (12.7, 12.9) | 11.3 (10.7, 11.9) | 3.7 (3.5, 3.8) | 5.4 (5.1, 5.7) | 4.1 (3.8, 4.4) |
|  | Adjusted^2^  (n = 450,002) | 13.0 (12.9, 13.1) | 11.0 (10.3, 11.6) | 3.7 (3.5, 3.9) | 5.2 (4.8, 5.6) | 4.2 (3.9, 4.5) |
| Physical health condition | Unadjusted  (n = 549,552) | 89,785 | 2,298 | 10,260 | 4,999 | 3,001 |
|  |  | 21.2 (21.0, 21.3) | 19.6 (18.9, 20.4) | 15.4 (15.2, 15.7) | 19.1 (18.6, 19.6) | 14.4 (14.0, 14.9) |
|  | Unadjusted  (n = 450,002) | 80,119 | 2,116 | 9,721 | 4,786 | 2,719 |
|  |  | 23.4 (23.3, 23.5) | 21.6 (20.8, 22.4) | 16.8 (16.5, 17.1) | 20.8 (20.3, 21.3) | 16.0 (15.4, 16.5) |
|  | Adjusted^2^  (n = 450,002) | 23.7 (23.6, 23.8) | 21.6 (20.8, 22.5) | 16.1 (15.7, 16.4) | 19.9 (19.2, 20.5) | 15.7 (15.1, 16.3) |

^1^ Adjusted for maternal age and previous pregnancy

^2^ Adjusted for maternal age, area-based level of deprivation and previous pregnancy

^3^ Adjusted for maternal age and area-based level of deprivation

**Table S10.** Unadjusted and adjusted prevalence of 10 prioritised preconception indicators by area-based level of deprivation

|  |  | **Area-based level of deprivation (quintiles)** | | | | |
| --- | --- | --- | --- | --- | --- | --- |
| **Preconception indicator** |  | **Most deprived**  **Quintile 1** | **Quintile 2** | **Quintile 3** | **Quintile 4** | **Least deprived Quintile 5** |
|  |  | n  % (95% CI) | n  % (95% CI) | n  % (95% CI) | n  % (95% CI) | n  % (95% CI) |
| ***Wider determinants of health*** |  |  |  |  |  |  |
| Complex social factors | Unadjusted  (n = 527,591) | 29,225 | 16,895 | 10,356 | 7,115 | 4,296 |
|  |  | 20.8 (20.6, 21.0) | 14.1 (13.9, 14.3) | 10.1 (10.0, 10.3) | 7.9 (7.7, 8.1) | 5.7 (5.5, 5.9) |
|  | Unadjusted  (n = 383833) | 20,026 | 12,758 | 7,713 | 5,305 | 3,251 |
|  |  | 20.0 (19.8, 20.3) | 14.4 (14.2, 14.6) | 10.3 (10.1, 10.5) | 8.1 (7.9, 8.3) | 5.9 (5.7, 6.1) |
|  | Adjusted  (n = 383833)^1^ | 17.2 (17.0, 17.4) | 13.8 (13.6, 14.0) | 11.2 (11.0, 11.4) | 9.9 (9.7, 10.1) | 8.4 (8.1, 8.6) |
| ***Reproductive health*** |  |  |  |  |  |  |
| Known previous obstetric complication | Unadjusted  (n = 329,228) | 24,415 | 18,040 | 14,714 | 12,470 | 11,055 |
|  |  | 26.4 (26.2, 26.7) | 24.2 (23.9, 24.5) | 23.8 (23.5, 24.2) | 22.9 (22.6, 23.3) | 23.9 (23.5, 24.3) |
|  | Unadjusted  (n = 290,394) | 21,759 | 16,114 | 13,160 | 11,199 | 10,032 |
|  |  | 26.9 (26.6, 27.2) | 24.6 (24.3, 25.0) | 24.1 (23.7, 24.5) | 23.3 (22.9, 23.7) | 24.3 (23.9, 24.7) |
|  | Adjusted^2^  (n = 290,394) | 27.2 (26.9, 27.6) | 24.8 (24.4, 25.1) | 24.5 (24.1, 24.9) | 23.4 (23.0, 23.8) | 23.6 (23.1, 24.1) |
| Previous pregnancy loss | Unadjusted  (n = 301,168) | 31,891 | 26,591 | 22,338 | 19,610 | 16,828 |
|  |  | 37.8 (37.5, 38.2) | 39.2 (38.8, 39.6) | 39.5 (39.1, 39.9) | 39.3 (38.8, 39.7) | 39.5 (39.1, 40.0) |
|  | Unadjusted  (n = 266,475) | 28,211 | 23,339 | 19,803 | 17,420 | 15,214 |
|  |  | 38.2 (37.8, 38.5) | 39.0 (38.6, 39.3) | 39.5 (39.1, 40.0) | 39.3 (39.1, 39.8) | 39.6 (39.1, 40.1) |
|  | Adjusted^2^  (n = 266,475) | 38.5 (38.1, 38.9) | 38.5 (38.1, 38.9) | 38.9 (38.5, 39.3) | 38.8 (38.3, 39.3) | 39.4 (38.9, 40.0) |
| ***Health behaviours and weight*** |  |  |  |  |  |  |
| Not taken folic acid supplementation before pregnancy | Unadjusted  (n = 488,987) | 106,823 | 86,979 | 67,399 | 53,766 | 40,681 |
|  |  | 83.0 (82.8, 83.2) | 75.5 (75.2, 75.7) | 70.3 (70.0, 70.6) | 65.5 (65.2, 65.9) | 60.6 (60.2, 60.9) |
|  | Unadjusted  (n = 360,993) | 79,634 | 65,192 | 50,216 | 39,510 | 29,899 |
|  |  | 83.8 (83.5, 84.0) | 76.5 (76.2, 76.7) | 70.9 (70.6, 71.2) | 65.5 (65.2, 65.9) | 60.3 (59.9, 60.8) |
|  | Adjusted^1^  (n = 360,993) | 81.3 (81.0, 81.6) | 75.8 (75.5, 76.1) | 72.1 (71.8, 72.4) | 68.4 (68.0, 68.8) | 64.7 (64.2, 65.1) |
| Smoking around conception | Unadjusted  (n = 604,514) | 44,429 | 28,663 | 20,156 | 14,833 | 9,521 |
|  |  | 27.8 (27.6, 28.0) | 20.8 (20.6, 21.0) | 17.3 (17.1, 17.5) | 14.4 (14.2, 14.7) | 10.9 (10.6, 11.1) |
|  | Unadjusted  (n = 435,059) | 33,170 | 21,356 | 14,755 | 10,646 | 6,423 |
|  |  | 28.7 (28.5, 29.0) | 21.2 (21.0, 21.5) | 17.6 (17.3, 17.8) | 14.6 (14.3, 14.8) | 10.4 (10.1, 10.6) |
|  | Adjusted^1^  (n = 435,059) | 29.4 (29.1, 29.7) | 21.7 (21.5, 22.0) | 17.6 (17.3, 17.8) | 15.1 (14.9, 15.4) | 12.1 (11.8, 12.4) |
| Overweight at booking (BMI 25 to 29.9 kg/m^2^) | Unadjusted  (n 496,331) | 36,043 | 31,997 | 27,077 | 23,681 | 19,976 |
|  |  | 28.4 (28.1, 28.6) | 28.3 (28.1, 28.6) | 28.1 (27.8, 28.4) | 27.6 (27.3, 27.9) | 26.9 (26.6, 27.2) |
|  | Unadjusted  (n = 368,258) | 27,159 | 24,220 | 20,189 | 17,512 | 14,649 |
|  |  | 28.6 (28.2, 28.8) | 28.6 (28.3, 28.8) | 28.3 (27.9, 28.5) | 27.7 (27.2, 27.9) | 27.0 (26.6, 27.3) |
|  | Adjusted^1^  (n = 368,258) | 28.3 (28.0, 28.6) | 28.4 (28.0, 28.7) | 28.3 (28.0, 28.7) | 27.8 (27.4, 28.1) | 27.1 (26.6, 27.5) |
| Obesity at booking (BMI ≥30 kg/m^2^) | Unadjusted  (n = 496,331) | 35,383 | 26,690 | 20,241 | 16,278 | 12,036 |
|  |  | 27.9 (27.6, 28.1) | 23.6 (23.4, 23.9) | 21.0 (20.8, 21.3) | 19.0 (18.7, 19.2) | 16.2 (16.0, 16.5) |
|  | Unadjusted  (n = 368,258) | 27,204 | 20,723 | 15,548 | 12,368 | 8,900 |
|  |  | 28.7 (28.4, 29.0) | 24.5 (24.2, 24.8) | 21.8 (21.5, 22.1) | 19.6 (19.3, 19.9) | 16.4 (16.1, 16.7) |
|  | Adjusted^1^  (n = 368,258) | 28.9 (28.5, 29.2) | 24.6 (24.3, 24.9) | 21.9 (21.6, 22.2) | 19.9 (19.6, 20.2) | 17.0 (16.6, 17.4) |
| ***Known pre-existing health conditions*** |  |  |  |  |  |  |
| Mental health condition | Unadjusted  (n = 652,880) | 19,107 | 14,174 | 11,466 | 9,458 | 6,768 |
|  |  | 10.9 (10.8, 11.1) | 9.6 (9.4, 9.7) | 9.2 (9.0, 9.3) | 8.5 (8.4, 8.7) | 7.2 (7.0, 7.3) |
|  | Unadjusted  (n = 450,002) | 15,766 | 11,219 | 9,095 | 7,540 | 5,437 |
|  |  | 13.2 (13.1, 13.4) | 10.8 (10.6, 11.0) | 10.5 (10.3, 10.7) | 9.9 (9.7, 10.2) | 8.4 (8.2, 8.7) |
|  | Adjusted^1^  (n = 450,002) | 13.8 (13.6, 14.1) | 11.2 (11.0, 11.4) | 10.3 (10.1, 10.5) | 9.7 (9.5, 9.9) | 8.5 (8.2, 8.7) |
| Physical health condition | Unadjusted  (n = 652,880) | 32,408 | 27,826 | 24,512 | 21,993 | 17,966 |
|  |  | 18.5 (18.3, 18.7) | 18.8 (18.6, 19.0) | 19.6 (19.4, 19.9) | 19.8 (19.6, 20.1) | 19.0 (18.8, 19.3) |
|  | Unadjusted  (n = 450,002) | 26,504 | 22,083 | 19,385 | 17,344 | 14,145 |
|  |  | 22.3 (22.0, 22.5) | 21.3 (21.0, 21.5) | 22.3 (22.0, 22.6) | 22.9 (22.6, 23.2) | 22.0 (21.6, 22.3) |
|  | Adjusted^1^  (n = 450,002) | 23.7 (23.4, 24.0) | 21.9 (21.6, 22.2) | 21.9 (21.7, 22.2) | 21.7 (21.4, 22.0) | 20.7 (20.4, 21.1) |

^1^ Adjusted for maternal age, ethnicity and previous pregnancy

^2^ Adjusted for maternal age and ethnicity

**Table S11.** Unadjusted and adjusted prevalence of 10 prioritised preconception indicators by previous pregnancy

|  |  | **Previous pregnancy** | | |
| --- | --- | --- | --- | --- |
| **Preconception indicator** |  | **Previous livebirth (with or without pregnancy loss)** | **Previous pregnancy loss (no livebirth)** | **No previous pregnancy** |
|  |  | n  % (95% CI) | n  % (95% CI) | n  % (95% CI) |
| ***Wider determinants of health*** |  |  |  |  |
| Living in most deprived area | Unadjusted  (n = 524,572) | 44,279 | 4,249 | 22,989 |
|  |  | 15.4 (15.2, 15.5) | 11.3 (11.0, 11.6) | 11.6 (11.4, 11.7) |
|  | Unadjusted  (n = 450,002) | 38,899 | 3,635 | 18,158 |
|  |  | 15.2 (15.1, 15.4) | 11.3 (10.9, 11.6) | 11.2 (11.0, 11.3) |
|  | Adjusted  (n = 450,002)^1^ | 16.3 (16.2, 16.5) | 10.7 (10.4, 11.0) | 10.0 (9.8, 10.1) |
| Complex social factors | Unadjusted  (n = 447,830) | 28,952 | 4,268 | 24,676 |
|  |  | 11.9 (11.8, 12.0) | 13.1 (12.8, 13.5) | 14.3 (14.2, 14.5) |
|  | Unadjusted  (n = 383,833) | 25,216 | 3,761 | 20,076 |
|  |  | 11.7 (11.5, 11.8) | 13.5 (13.1, 13.9) | 14.3 (14.2, 14.5) |
|  | Adjusted  (n = 383,833)^2^ | 13.7 (13.5, 13.8) | 12.9 (12.5, 13.3) | 11.1 (10.9, 11.2) |
| ***Health behaviours and weight*** |  |  |  |  |
| Not taken folic acid supplementation before pregnancy | Unadjusted  (n = 422,534) | 178,559 | 19,422 | 111,462 |
|  |  | 77.1 (76.9, 77.3) | 64.8 (64.3, 65.4) | 69.3 (69.0, 69.5) |
|  | Unadjusted  (n = 360,993) | 157,622 | 16,543 | 90,286 |
|  |  | 77.0 (76.8, 77.1) | 64.6 (64.1, 65.2) | 69.1 (68.9, 69.4) |
|  | Adjusted^2^  (n = 360,993) | 78.0 (77.8, 78.2) | 63.6 (63.1, 64.2) | 66.5 (66.2, 66.7) |
| Smoking around conception | Unadjusted  (n = 504,934) | 56,397 | 7,487 | 33,577 |
|  |  | 20.2 (20.1, 20.4) | 20.7 (20.2, 21.1) | 17.7 (17.5, 17.8) |
|  | Unadjusted  (n = 435,059) | 51,208 | 6,596 | 28,546 |
|  |  | 20.6 (20.5, 20.8) | 21.1 (20.6, 21.5) | 18.3 (18.1, 18.5) |
|  | Adjusted^2^  (n = 435,059) | 21.9 (21.8, 22.1) | 19.6 (19.2, 20.0) | 16.4 (16.2, 16.6) |
| Overweight at booking (BMI 25 to 29.9 kg/m^2^) | Unadjusted  (n = 421,712) | 68,457 | 8,876 | 40,789 |
|  |  | 29.1 (28.9, 29.3) | 28.1 (27.6, 28.6) | 26.3 (26.1, 26.6) |
|  | Unadjusted  (n = 368,258) | 61,592 | 7,763 | 34,374 |
|  |  | 29.1 (28.9, 29.3) | 28.3 (27.7, 28.8) | 26.6 (26.4, 26.8) |
|  | Adjusted^2^  (n = 368,258) | 28.6 (28.4, 28.8) | 28.5 (27.9, 29.0) | 27.0 (26.7, 27.2) |
| Obesity at booking (BMI ≥30 kg/m^2^) | Unadjusted  (n = 421,712) | 59,521 | 6,701 | 28,524 |
|  |  | 25.3 (25.1, 25.5) | 21.2 (20.7, 21.6) | 18.4 (18.2, 18.6) |
|  | Unadjusted  (n = 368,258) | 54,312 | 5,902 | 24,529 |
|  |  | 25.7 (25.5, 25.8) | 21.5 (21.0, 22.0) | 19.0 (18.8, 19.2) |
|  | Adjusted^2^  (n = 368,258) | 25.5 (25.3, 25.7) | 21.5 (21.0, 22.0) | 19.4 (19.2, 19.6) |
| ***Known pre-existing health conditions*** |  |  |  |  |
| Mental health condition | Unadjusted  (n = 524,572) | 32,777 | 4,674 | 17,766 |
|  |  | 11.4 (11.3, 11.5) | 12.4 (12.1, 12.8) | 8.9 (8.8, 9.1) |
|  | Unadjusted  (n = 450,002) | 29,869 | 4,043 | 15,145 |
|  |  | 11.7 (11.6, 11.8) | 12.5 (12.2, 12.9) | 9.3 (9.2, 9.5) |
|  | Adjusted^2^  (n = 450,002) | 12.0 (11.8, 12.1) | 12.1 (11.7, 12.4) | 8.9 (8.8, 9.0) |
| Physical health condition | Unadjusted  (n = 524,572) | 64,443 | 9,189 | 38,576 |
|  |  | 22.4 (22.2, 22.5) | 24.5 (24.0, 24.9) | 19.4 (19.2, 19.6) |
|  | Unadjusted  (n = 450,002) | 58,930 | 8,006 | 32,525 |
|  |  | 23.1 (22.9, 23.2) | 24.8 (24.3, 25.3) | 20.0 (19.8, 20.2) |
|  | Adjusted^2^  (n = 450,002) | 22.3 (22.1, 22.5) | 25.2 (24.7, 25.7) | 21.1 (20.9, 21.3) |

^1^ Adjusted for maternal age and ethnicity

^2^ Adjusted for maternal age, ethnicity and area-based level of deprivation

**Table S12.** Proportions of missing data of preconception indicators by maternal age

|  | **Overall** | **Maternal age** | | | | | | |
| --- | --- | --- | --- | --- | --- | --- | --- | --- |
| **Preconception indicator variable** | (N = 652,880) | **<20**  (n = 24,675) | **20-24**  (n = 97,178) | **25-29**  (n = 184,186) | **30-34**  (n = 207,171) | **35-39**  (n = 113,458) | **40+**  (n = 26,203) | **Missing**  (n = 9) |
| **Ethnicity** |  |  |  |  |  |  |  |  |
| % not stated | 9.5 | 9.0 | 8.8 | 9.5 | 10.0 | 9.5 | 9.4 | 22.0 |
| % not stated or missing | 15.8 | 15.4 | 15.4 | 16.0 | 16.3 | 15.4 | 15.2 | 33.1 |
| **Employment** |  |  |  |  |  |  |  |  |
| % not stated | 7.4 | 9.3 | 8.3 | 7.7 | 7 | 6.7 | 6.5 | 22.2 |
| % not stated or missing | 27.7 | 32.2 | 29.5 | 28.3 | 26.8 | 26.2 | 26.2 | 33.3 |
| **Complex social factors** |  |  |  |  |  |  |  |  |
| % missing | 19.2 | 18.8 | 20.7 | 19.8 | 18.8 | 18 | 17.8 | 11.1 |
| **English as first language** |  |  |  |  |  |  |  |  |
| % not stated | 8.1 | 8.3 | 8.2 | 8.1 | 8.1 | 8 | 7.8 | - |
| % not stated or missing | 23.8 | 25.3 | 24.6 | 24.4 | 23.5 | 22.7 | 22.4 | 11.1 |
| **Support status** |  |  |  |  |  |  |  |  |
| % not stated | 6.9 | 7.3 | 6.7 | 6.8 | 7.1 | 6.9 | 6.6 | - |
| % not stated or missing | 31.1 | 32.8 | 31.0 | 30.9 | 31.1 | 30.9 | 31.2 | 22.2 |
| **Previous pregnancy loss** |  |  |  |  |  |  |  |  |
| % missing | 23.4 | 28.7 | 25.1 | 23.9 | 22.9 | 21.5 | 20.8 | 15.4 |
| **Folic acid supplementation** |  |  |  |  |  |  |  |  |
| % not stated | 6.1 | 6.1 | 5.5 | 5.6 | 6.4 | 6.9 | 6.7 | - |
| % not stated or missing | 25.1 | 26.0 | 24.8 | 24.6 | 25.1 | 25.6 | 26.1 | 22.2 |
| **Smoking status** |  |  |  |  |  |  |  |  |
| % missing | 7.4 | 8.4 | 7.8 | 7.6 | 7.2 | 7.0 | 7.2 | 11.1 |
| **Body Mass Index** |  |  |  |  |  |  |  |  |
| % missing | 24.0 | 26.0 | 24.6 | 24.3 | 23.7 | 23.3 | 23.4 | 53.2 |

Not stated: Person asked but declined to provide a response.

**Table S13.** Proportions of missing data of preconception indicators by ethnicity

|  | **Overall** |  |  | **Ethnicity** |  |  |  |  |
| --- | --- | --- | --- | --- | --- | --- | --- | --- |
| **Preconception indicator** | (N = 652,880) | **White**  (n = 424,453) | **Mixed**  (n = 11,695) | **Asian**  (n = 66,444) | **Black**  (n = 26,172) | **Other**  (n = 20,788) | **Missing**  (n = 41,286) | **Not stated**  (n = 62,042) |
| **Employment** |  |  |  |  |  |  |  |  |
| % not stated | 7.4 | 7.3 | 5.7 | 5.6 | 5.7 | 10.2 | 14.4 | 5.6 |
| % not stated or missing | 27.7 | 27.5 | 23.3 | 21.6 | 19.6 | 25.3 | 39.4 | 33 |
| **Complex social factors** |  |  |  |  |  |  |  |  |
| % missing | 19.2 | 19.5 | 16.7 | 14.8 | 17.0 | 13.5 | 23.5 | 22.2 |
| **English as first language** |  |  |  |  |  |  |  |  |
| % not stated | 8.1 | 7.7 | 7.2 | 7.3 | 6.6 | 6.2 | 11.2 | 10.6 |
| % not stated or missing | 23.8 | 22.4 | 23.1 | 22.1 | 18.8 | 18.8 | 33.2 | 32.7 |
| **Support status** |  |  |  |  |  |  |  |  |
| % not stated | 6.9 | 6.7 | 6.5 | 6.6 | 5.1 | 6.9 | 9.9 | 7.4 |
| % not stated or missing | 31.1 | 30.2 | 30.5 | 27.5 | 25.8 | 28 | 38.1 | 39.5 |
| **Previous pregnancy loss** |  |  |  |  |  |  |  |  |
| % missing | 23.4 | 23.1 | 20.0 | 17.0 | 15.4 | 21.7 | 30.5 | 32.0 |
| **Folic acid supplementation** |  |  |  |  |  |  |  |  |
| % not stated | 6.1 | 6.1 | 6.1 | 5.6 | 4.3 | 5 | 6.8 | 7.3 |
| % not stated or missing | 25.1 | 23.8 | 26 | 25.5 | 24.2 | 26.2 | 27.9 | 31.3 |
| **Smoking status** |  |  |  |  |  |  |  |  |
| % missing | 7.4 | 6.5 | 5.6 | 6.8 | 5.2 | 6.1 | 15.4 | 11 |
| **Body Mass Index** |  |  |  |  |  |  |  |  |
| % missing | 24.0 | 22.7 | 20.7 | 20.1 | 19.4 | 21.6 | 29.7 | 37.1 |

Not stated: Person asked but declined to provide a response.

**Table S14.** Proportions of missing data of preconception indicators by area-based level of deprivation

|  | **Overall** | **Area-based level of deprivation** | | | | |
| --- | --- | --- | --- | --- | --- | --- |
| **Preconception indicator** | (N = 652,880) | **Most deprived**  **Quintile 1**  (n = 174,925) | **Quintile 2**  (n = 147,740) | **Quintile 3**  (n = 124,847) | **Quintile 4**  (n = 110,983) | **Least deprived Quintile 5**  (n = 94,385) |
| **Ethnicity** |  |  |  |  |  |  |
| % not stated | 9.5 | 9.5 | 8.9 | 9.3 | 10.3 | 9.8 |
| % not stated or missing | 15.8 | 16.1 | 15.6 | 15.8 | 16.4 | 15.1 |
| **Employment** |  |  |  |  |  |  |
| % not stated | 7.4 | 8.9 | 6.1 | 6.5 | 7.5 | 7.6 |
| % not stated or missing | 27.7 | 30.8 | 23.7 | 25.6 | 28.6 | 29.8 |
| **Complex social factors** |  |  |  |  |  |  |
| % missing | 19.2 | 19.8 | 18.9 | 18.2 | 19.1 | 19.9 |
| **English as first language** |  |  |  |  |  |  |
| % not stated | 8.1 | 7.7 | 7.1 | 7.9 | 8.5 | 10.2 |
| % not stated or missing | 23.8 | 25.0 | 21.0 | 22.6 | 24.8 | 26.5 |
| **Support status** |  |  |  |  |  |  |
| % not stated | 6.9 | 5.6 | 5.5 | 6.9 | 8.1 | 10.3 |
| % not stated or missing | 31.1 | 29.6 | 27.9 | 30.7 | 33.4 | 36.7 |
| **Previous pregnancy loss** |  |  |  |  |  |  |
| % missing | 23.4 | 23.9 | 21.5 | 22.7 | 24.3 | 25.4 |
| **Folic acid supplementation** |  |  |  |  |  |  |
| % not stated | 6.1 | 4.6 | 4.3 | 5.7 | 7.7 | 10.7 |
| % not stated or missing | 25.1 | 26.5 | 22 | 23.2 | 26 | 28.9 |
| **Smoking status** |  |  |  |  |  |  |
| % missing | 7.4 | 8.6 | 6.8 | 6.7 | 7.5 | 7.1 |
| **Body Mass Index** |  |  |  |  |  |  |
| % missing | 24.0 | 24.7 | 22.4 | 24.0 | 24.9 | 24.5 |

Not stated: Person asked but declined to provide a response.

**Table S15.** Proportions of missing data of preconception indicators by previous pregnancy

|  | **Overall** | **Previous pregnancy** | | | |
| --- | --- | --- | --- | --- | --- |
| **Preconception indicator** | (N = 652,880) | **Previous livebirth (with or without pregnancy loss)**  (n = 288,073) | **Previous pregnancy loss (no livebirth)**  (n = 41,154) | **No previous pregnancy**  (n = 198,949) | **Missing**  (n = 124,704) |
| **Ethnicity** |  |  |  |  |  |
| % not stated | 9.5 | 6.7 | 9.2 | 10.9 | 13.8 |
| % not stated or missing | 15.8 | 11.4 | 14.6 | 18.3 | 22.5 |
| **Employment** |  |  |  |  |  |
| % not stated | 7.4 | 5.4 | 6.2 | 4.1 | 17.7 |
| % not stated or missing | 27.7 | 18.7 | 17.4 | 19.8 | 64.6 |
| **Complex social factors** |  |  |  |  |  |
| % missing | 19.2 | 15.6 | 17.0 | 13.5 | 37.3 |
| **English as first language** |  |  |  |  |  |
| % not stated | 8.1 | 4.7 | 4.8 | 7.0 | 18.7 |
| % not stated or missing | 23.8 | 15.0 | 14.7 | 19.9 | 53.2 |
| **Support status** |  |  |  |  |  |
| % not stated | 6.9 | 4.4 | 6.0 | 4.9 | 16.2 |
| % not stated or missing | 31.1 | 22.9 | 25.4 | 23.7 | 63.6 |
| **Folic acid supplementation** |  |  |  |  |  |
| % not stated | 6.1 | 4.4 | 6.0 | 4.9 | 16.2 |
| % not stated or missing | 25.1 | 22.9 | 25.4 | 23.7 | 63.6 |
| **Smoking status** |  |  |  |  |  |
| % missing | 7.4 | 3.3 | 3.9 | 4.5 | 22.8 |
| **Body Mass Index** |  |  |  |  |  |
| % missing | 24.0 | 17.3 | 16.5 | 21.8 | 45.7 |

Not stated: Person asked but declined to provide a response.

**References**

1. Schoenaker DA, Stephenson J, Connolly A, Shillaker S, Fishburn S, Barker M, et al. Characterising and monitoring preconception health in England: a review of national population-level indicators and core data sources. J Dev Orig Health Dis. 2022;13(2):137-50.
2. NHS Digital. Maternity Services Dataset v1.5 archived guidance documents. Available from: <https://digital.nhs.uk/data-and-information/data-collections-and-data-sets/data-sets/maternity-services-data-set/archived-guidance-documents>. (accessed 27 October 2022).
